# Hypoalbuminemia affects one third of acute pancreatitis patients and is independently associated with severity and mortality

**DOI:** 10.1038/s41598-021-03449-8

**Published:** 2021-12-17

**Authors:** Klementina Ocskay, Zsófia Vinkó, Dávid Németh, László Szabó, Judit Bajor, Szilárd Gódi, Patrícia Sarlós, László Czakó, Ferenc Izbéki, József Hamvas, Mária Papp, Márta Varga, Imola Török, Artautas Mickevicius, Ville Sallinen, Elena Ramirez Maldonado, Shamil Galeev, Alexandra Mikó, Bálint Erőss, Marcell Imrei, Péter Jenő Hegyi, Nándor Faluhelyi, Orsolya Farkas, Péter Kanizsai, Attila Miseta, Tamás Nagy, Roland Hágendorn, Zsolt Márton, Zsolt Szakács, Andrea Szentesi, Péter Hegyi, Andrea Párniczky

**Affiliations:** 1 Institute for Translational Medicine, Szentágothai Research Centre, Medical School, University of Pécs, Pécs, Hungary; 2 Centre for Translational Medicine, Semmelweis University, Budapest, Hungary; 3 Department of Medicine, Centre for Translational Medicine, University of Szeged, Szeged, Hungary; 4 Division of Gastroenterology, First Department of Medicine, Medical School, University of Pécs, Pécs, Hungary; 5 Department of Medicine, University of Szeged, Szeged, Hungary; 6 Szent György University Teaching Hospital of Fejér County, Székesfehérvár, Hungary; 7 Péterfy Hospital, Budapest, Hungary; 8 Department of Internal Medicine, Division of Gastroenterology, University of Debrecen, Debrecen, Hungary; 9 Dr. Réthy Pál Hospital, Békéscsaba, Hungary; 10 County Emergency Clinical Hospital – Gastroenterology and University of Medicine, Pharmacy, Sciences and Technology, Targu Mures, Romania; 11 Vilnius University Hospital Santaros Clinics, Vilnius, Lithuania; 12 Clinics of Abdominal Surgery, Nephrourology and Gastroenterology, Faculty of Medicine, Vilnius University, Vilnius, Lithuania; 13 Department of Transplantation and Liver Surgery, Helsinki University Hospital and University of Helsinki, Helsinki, Finland; 14 General Surgery, Consorci Sanitari del Garraf, Sant Pere de Ribes, Barcelona, Spain; 15 North-Western State Medical University, Saint-Petersburg, Russia; 16 Division of Pancreatic Diseases, Heart and Vascular Center, Semmelweis University, Budapest, Hungary; 17 Department of Medical Imaging, Medical School, University of Pécs, Pécs, Hungary; 18 Department of Emergency Medicine, Medical School, University of Pécs, Pécs, Hungary; 19 Department of Laboratory Medicine, Medical School, University of Pécs, Pécs, Hungary; 20 First Department of Medicine, Medical School, University of Pécs, Pécs, Hungary; 21 Heim Pál National Paediatric Institute, Budapest, Hungary

**Keywords:** Pancreatitis, Acute pancreatitis, Biomarkers

## Abstract

The incidence and medical costs of acute pancreatitis (AP) are on the rise, and severe cases still have a 30% mortality rate. We aimed to evaluate hypoalbuminemia as a risk factor and the prognostic value of human serum albumin in AP. Data from 2461 patients were extracted from the international, prospective, multicentre AP registry operated by the Hungarian Pancreatic Study Group. Data from patients with albumin measurement in the first 48 h (n = 1149) and anytime during hospitalization (n = 1272) were analysed. Multivariate binary logistic regression and Receiver Operator Characteristic curve analysis were used. The prevalence of hypoalbuminemia (< 35 g/L) was 19% on admission and 35.7% during hospitalization. Hypoalbuminemia dose-dependently increased the risk of severity, mortality, local complications and organ failure and is associated with longer hospital stay. The predictive value of hypoalbuminemia on admission was poor for severity and mortality. Severe hypoalbuminemia (< 25 g/L) represented an independent risk factor for severity (OR 48.761; CI 25.276–98.908) and mortality (OR 16.83; CI 8.32–35.13). Albumin loss during AP was strongly associated with severity (p < 0.001) and mortality (p = 0.002). Hypoalbuminemia represents an independent risk factor for severity and mortality in AP, and it shows a dose-dependent relationship with local complications, organ failure and length of stay.

## Introduction

Acute pancreatitis is a common gastroenterological disorder, with rising incidence and high medical costs. The commonly used revised Atlanta Classification distinguishes between mild, moderate and severe disease based on the development and duration of organ failure^1^. As the mortality rate can reach 30% in severe cases, identifying risk factors and potential therapeutic targets is of utmost importance.

Human serum albumin is the most abundant protein in human serum with a very diverse role. Although this hypothesis has been contradicted by recent data, declining albumin levels during inflammation have long prompted physicians to underestimate its contribution to maintaining homeostasis during inflammation. However, albumin plays a pivotal role in maintaining the plasma redox state^2^, and its scavenging activity is likely to influence vascular resistance through the regulation of nitric oxide levels^3^. Furthermore, low albumin levels result in dilution and increased drug clearance, ultimately causing sub-optimal treatment^4^.

Small retrospective cohort studies have shown that hypoalbuminemia is an independent risk factor for severe AP and in-hospital mortality in adults and children^5,6^. Serum albumin has been reported to be associated with persistent organ failure and prolonged hospital stay^7^. However, whether albumin is only a marker or there is a cause-effect relationship between hypoalbuminemia and disease severity and mortality should be further evaluated.

While comprehensive analyses are missing on AP patients with hypoalbuminemia and albumin loss in AP, we aimed to evaluate (1) on-admission and in-hospital hypoalbuminemia as a risk factor in AP, (2) the prognostic potential of human serum albumin, (3) whether there is a dose-dependent relationship between albumin level and disease outcomes and (4) the relation of albumin loss to severity and mortality.

We found evidence that AP patients with < 25 g/L serum albumin anytime during hospitalization have a 16.8-fold higher risk of death and 48.8-fold higher risk of severe AP than patients with normal albumin levels. We also observed that albumin loss during AP is associated with severity and mortality. These data highlight the unmet need for randomized controlled trials focusing on albumin replacement.

## Results

### One in every five patients suffering from acute pancreatitis has hypoalbuminemia on admission

Nineteen percent of patients (n = 218/1149) presented with hypoalbuminemia (< 35 g/L). 12.4% of patients were admitted with 30–34.99 g/L albumin levels (Group 5), whereas 4.4% and 2.2% of patients had 25–29.99 g/L (Group 6) and < 25 g/L (Group 7) on-admission albumin levels (Sup. Fig. S3).

### Older age, lower body mass index, abdominal guarding on physical examination and non-biliary aetiology are associated with on-admission hypoalbuminemia

Hypoalbuminemia was associated with older age (average 59.7 ± 18.0 and 56.0 ± 16.1 years; p = 0.005, Sup. Fig. S3). Males were overrepresented in the analysed cohort (57%) and all subgroups (Sup. Fig. S3). Although biliary aetiology was the most frequent in all subgroups, significantly fewer patients had biliary aetiology (34.4% vs 42.2%; p = 0.042) in the low albumin group, and a tendency of more alcoholic episodes (24.3% and 19%; p = 0.096) was seen (Sup. Fig. S3).

Significantly lower body mass index (average 28.23 and 27.23; p = 0.012) was found in the low albumin group compared to the normal albumin group (Sup. Fig. S4). Diabetes mellitus (22.6% vs. 19.3%; p = 0.318) and chronic pancreatitis (7.3% vs. 6.1%, p = 0.507) were overrepresented in patients with hypoalbuminemia; however, fewer patients with hypoalbuminemia had recurrent AP (17.4% vs. 21.9%, p = 0.144) (Sup. Fig. S4).

As regards the signs and symptoms, fewer hypoalbuminemia patients presented with abdominal pain (94.9% and 99.2%; p < 0.001) and more with abdominal guarding (27.2% and 19.9%; p = 0.023) (Fig. S5). General signs, such as duration and intensity of abdominal pain, abdominal tenderness, nausea and vomiting, did not differ significantly. Hypoalbuminemia was associated with a dose-dependent increase in heart rate and a decrease in systolic and diastolic blood pressure on admission (Sup. Fig. S5).

The fulfilment of diagnostic criteria differed significantly (p < 0.001) among the low and normal albumin groups on admission. Low albumin patients were less likely to present with pancreatic enzyme elevation, abdominal pain and characteristic imaging findings at the same time (42.7% versus 58.4%) (Sup. Table S2).

### On-admission hypoalbuminemia is dose-dependently associated with elevated CRP and PCT levels in AP

The low albumin group had significantly lower serum amylase (p < 0.001) and lipase (p = 0.002) levels on admission. An increase in dose-dependent C-reactive protein (CRP) (p < 0.001) and procalcitonin (PCT) (p < 0.001) was observed in the lower albumin groups. White blood cell count (WBC) (p = 0.017) levels were also significantly elevated in the low albumin group (Figs. S6–S7). As regards laboratory markers of renal function, hypoalbuminemia patients had significantly higher blood urea nitrogen (BUN) (p = 0.002) and creatinine (p = 0.002) levels and a lower estimated glomerular filtration rate (eGFR) (p < 0.001) (Sup. Figs. S8–S9). Liver enzymes and total bilirubin levels did not differ between the low and normal albumin groups, but hypoalbuminemia was associated with higher direct bilirubin levels (p = 0.005) and a higher international normalized ratio (INR) (p < 0.001) (Sup. Figs. S10–S13). Haematological parameters, lipids, ions and glucose levels are shown in Supplementary Figs. S14–S17.

### On-admission hypoalbuminemia is dose-dependently associated with complications, severity and mortality in AP

**Figure 1:**
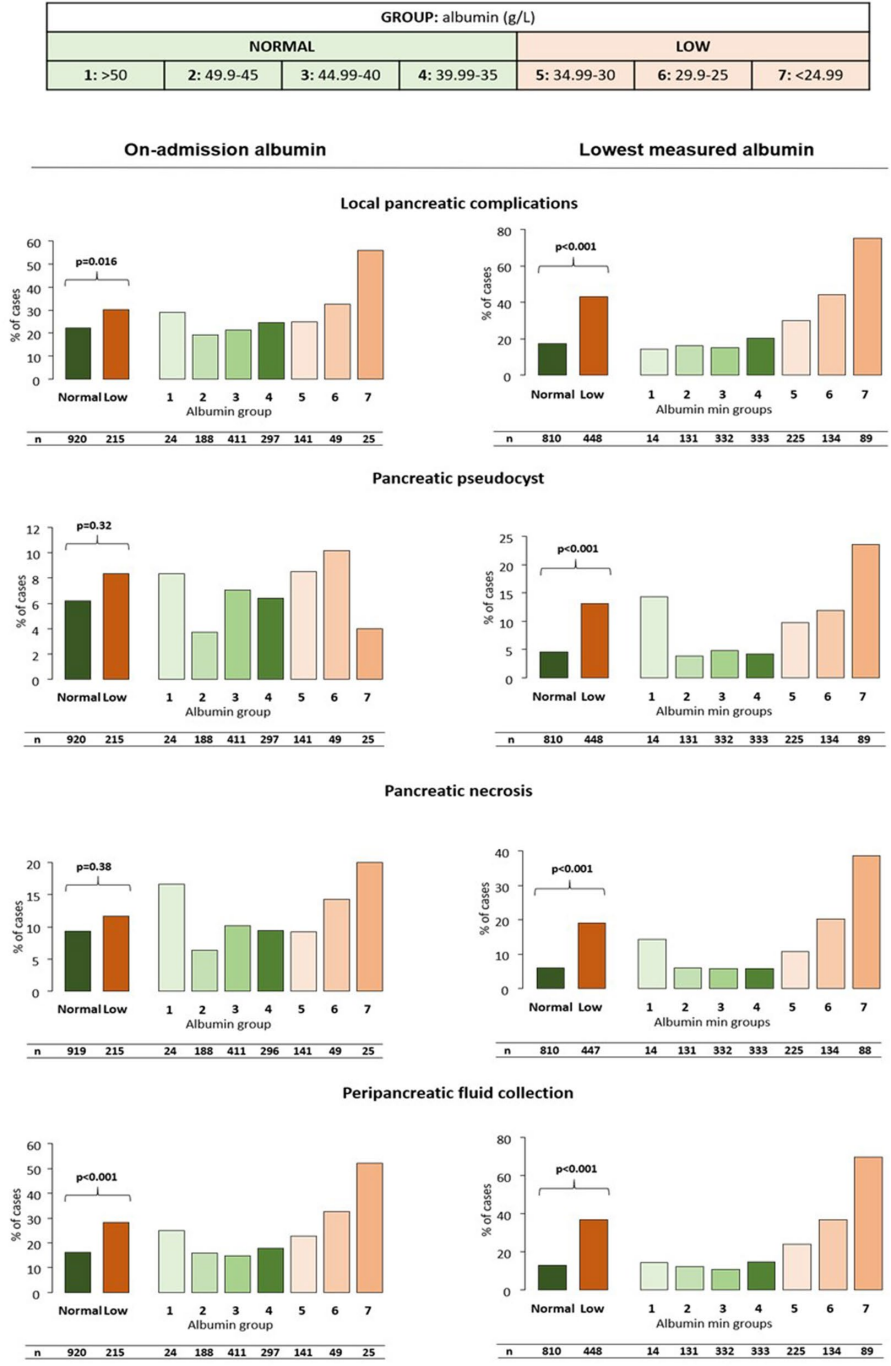
Relation between albumin level and local complications, as defined by the Revised Atlanta Criteria in acute pancreatitis. All types of local complications were significantly more frequent in the low albumin group. A dose-dependent increase was seen in the rate of local complications and peripancreatic fluid collection in both cohorts and in pancreatic necrosis and pseudocyst in the lowest measured albumin cohort. P < 0.05 is considered significant. Patients with albumin levels < 35 g/L were included in the low albumin group (Groups 5–7).

**Figure 2:**
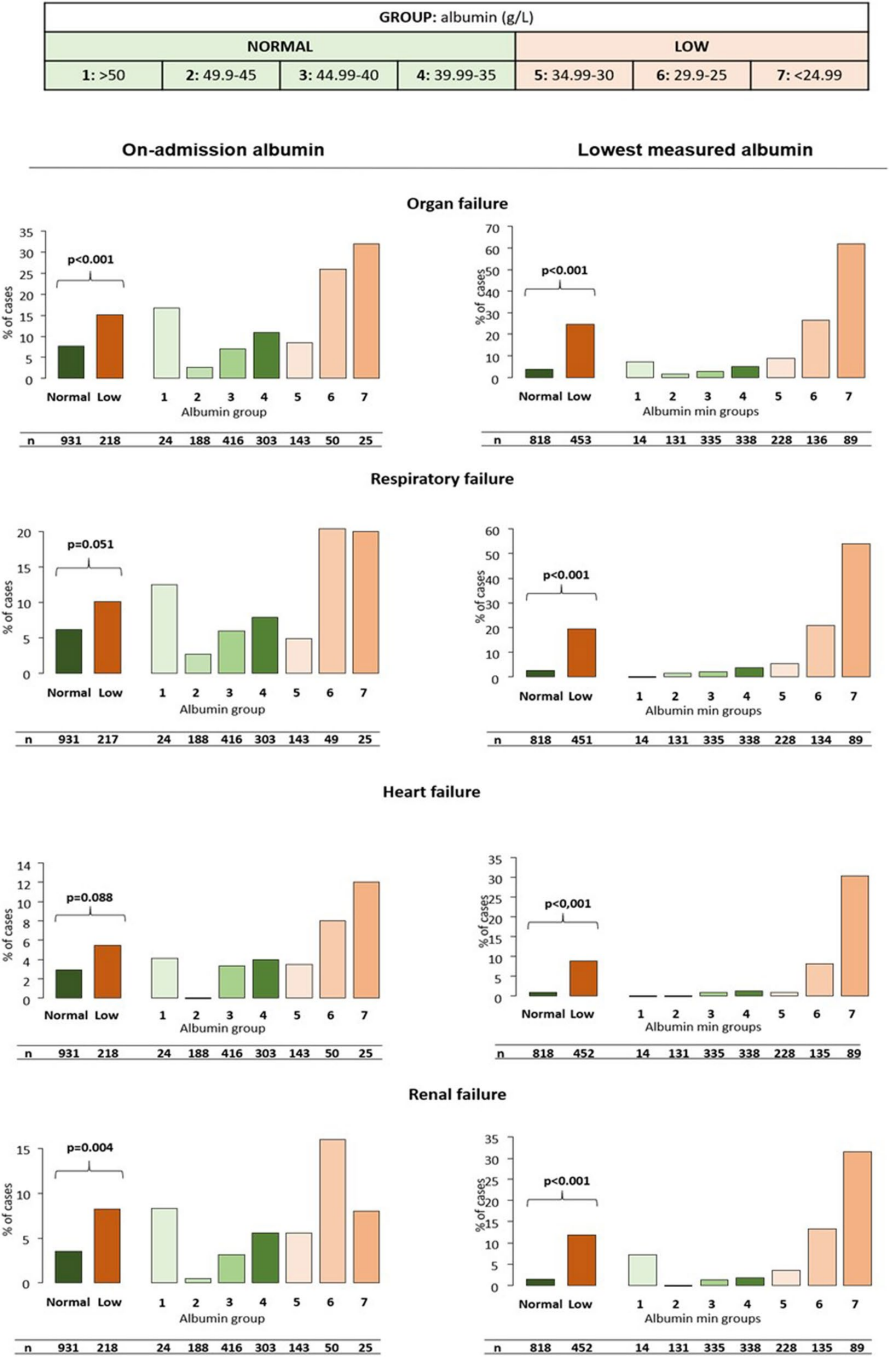
Relation between albumin level and organ failure, as defined by the Revised Atlanta Criteria in acute pancreatitis. Significantly more patients developed organ failure in the low albumin group in both cohorts. A dose-dependent increase was seen in the case of all analyses in the lowest measured albumin cohort. Heart failure was dose-dependently increased in the on-admission cohort as well. P < 0.005 is considered significant.

Significantly more patients developed local complications and organ failure in the low albumin group (p = 0.016 and p < 0.001, respectively) (Figs. 1, 2). Lower albumin levels correlated with a higher rate of peripancreatic fluid collection and respiratory failure (p < 0.001 and p = 0.051). The rate of pancreatic necrosis, pseudocyst or heart failure did not differ significantly between the groups.

**Figure 3:**
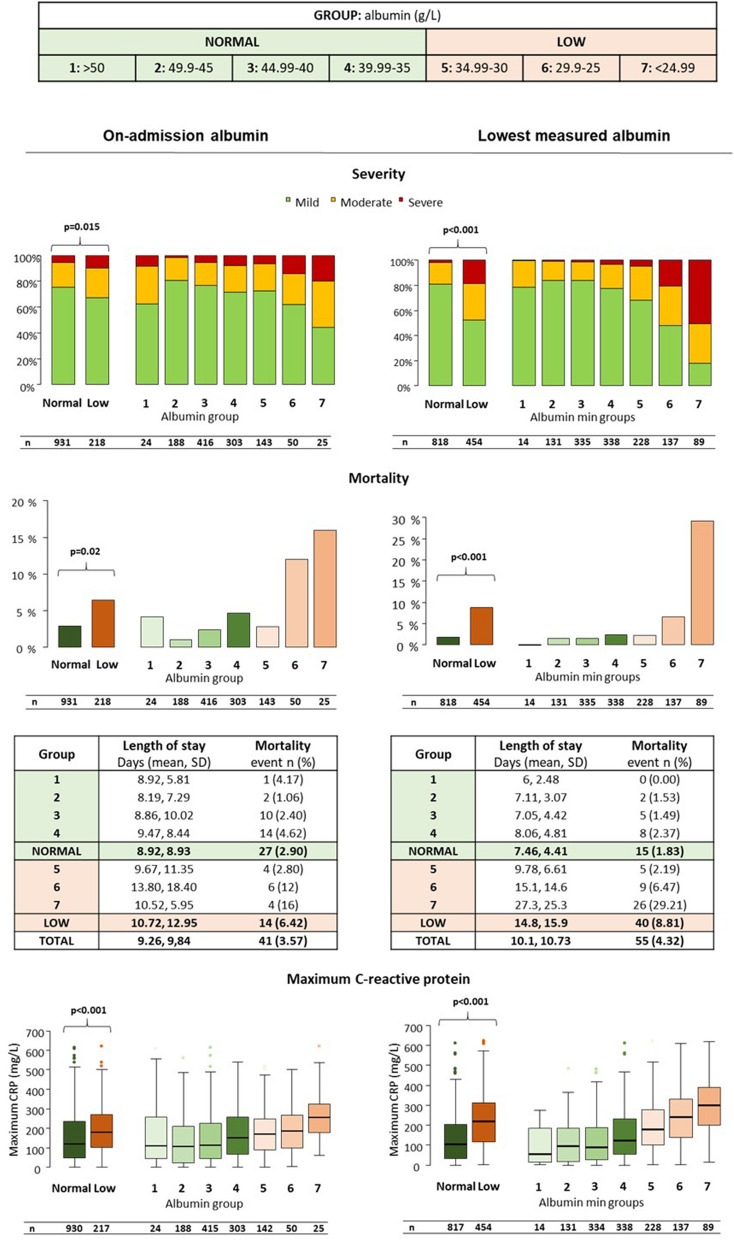
Relation between albumin level and disease severity, mortality, length of stay and maximum C-reactive protein level in acute pancreatitis. Severity, mortality, length of stay and maximum C-reactive protein levels were significantly and dose-dependently associated with hypoalbuminemia in both cohorts. P < 0.05 is considered significant.

Most importantly, hypoalbuminemia was associated with increased mortality (p = 0.020), disease severity (p = 0.015) and hospital stay (p = 0.025) (Fig. 3). Groups 6 and 7 had significantly higher mortality (p = 0.005 and p = 0.007, respectively) and severity (p = 0.028 and p < 0.001, respectively) compared to the normal group. Maximum CRP levels during the course of AP significantly and dose-dependently increased with the degree of serum albumin (p < 0.001, Fig. 3).

### On-admission hypoalbuminemia is an independent risk factor for severity and mortality, with an odds ratio of up to 5.3 for mortality in acute pancreatitis

Age, hypertriglyceridemia-induced (with or without concomitant alcoholic aetiology) and idiopathic AP were independently associated with mortality. Severe on-admission hypoalbuminemia proved to be an independent risk factor for mortality with an OR of 3.782 (CI 1.313–9.462) in Group 6 (< 30 g/L) and an OR of 5.256 (CI 1.389–16.112) in Group 7 (< 25 g/L) (Table 1). Albumin levels were examined with a 35 g/L cut-off in a separate analysis, which found an independent relation between hypoalbuminemia and mortality (OR 2.070; CI 1.021–4.033; Supplementary Table S3). Age, hypertriglyceridemia-induced AP, and, among the multifactorial aetiologies, a combination of hypertriglyceridemia and alcohol were independent risk factors for disease severity. On-admission albumin levels < 25 g/L were independently associated with severe AP (OR 3.620; CI 1.128–9.978; Table 1).

**Table 1:** Multivariate logistic regression analysis on the prognostic role of on-admission hypoalbuminemia in acute pancreatitis.

Predictor	β	SE	OR	95% CI	p
**On-admission albumin (n = 1149)—mortality**					
On-admission albumin level					
30–34.99 g/L (vs. ≥ 35 g/L)	− 0.108	0.553	0.898	0.259–2.390	0.845
25–29.99 g/L (vs. ≥ 35 g/L)	1.330	0.496	3.782	1.313–9.462	**0.007**
< 25 g/L (vs. ≥ 35 g/L)	1.659	0.611	5.256	1.389–16.112	**0.007**
Age					
Per years	0.037	0.012	1.037	0.014–1.063	**0.003**
Gender					
Female (vs. male)	− 0.222	0.370	0.801	0.383–1.648	0.548
Aetiology					
Alcohol (vs. biliary)	0.669	0.554	1.952	0.636–5.725	0.227
HTG (vs. biliary)	1.669	0.747	5.304	1.037–21.022	**0.025**
Biliary + alcohol (vs. biliary)	1.234	1.100	3.436	0.178–20.816	0.262
Biliary + HTG (vs. biliary)	− 12.903	783.282	–	–	0.987
Alcohol + HTG (vs. biliary)	1.781	0.768	5.938	1.123–24.693	**0.020**
Idiopathic (vs. biliary)	1.119	0.427	3.061	1.330–7.223	**0.009**
Other (vs. biliary)	0.010	0.790	1.010	0.152–3.964	0.990
**On-admission albumin (n = 1149)—severity**					
On-admission albumin					
30–34.99 g/L (v. ≥ 35 g/L)	0.029	0.383	1.030	0.457–2.086	0.939
25–29.99 g/L (v. ≥ 35 g/L)	0.829	0.449	2.292	0.882–5.238	0.065
< 25 g/L (v. ≥ 35 g/L)	1.286	0.548	3.620	1.118–9.968	**0.019**
Age					
Per years	0.040	0.010	1.041	1.022–1.061	** < 0.001**
Gender					
Female (vs. male)	− 0.183	0.281	0.830	0.478–1.442	0.515
Aetiology					
Alcohol (vs. biliary)	0.522	0.420	1.685	0.751–3.673	0.195
HTG (vs. biliary)	1.712	0.546	5.543	1.776–15.536	**0.002**
Biliary + alcohol (vs. biliary)	1.056	0.802	2.874	0.426–11.572	0.188
Biliary + HTG (vs. biliary)	− 13.792	785.525	–	–	0.986
Alcohol + HTG (vs. biliary)	1.316	0.632	3.727	0.952–11.941	**0.037**
Idiopathic (vs. biliary)	0.536	0.330	1.709	0.884–3.247	0.104
Other (vs. biliary)	− 0.475	0.629	0.622	0.145–1.852	0.450

*HTG* hypertriglyceridemia, *β* β coefficient, *SE* standard error *OR* odds ratio, *CI* confidence interval.

### On-admission albumin levels alone have poor predictive value in AP

**Figure 4:**
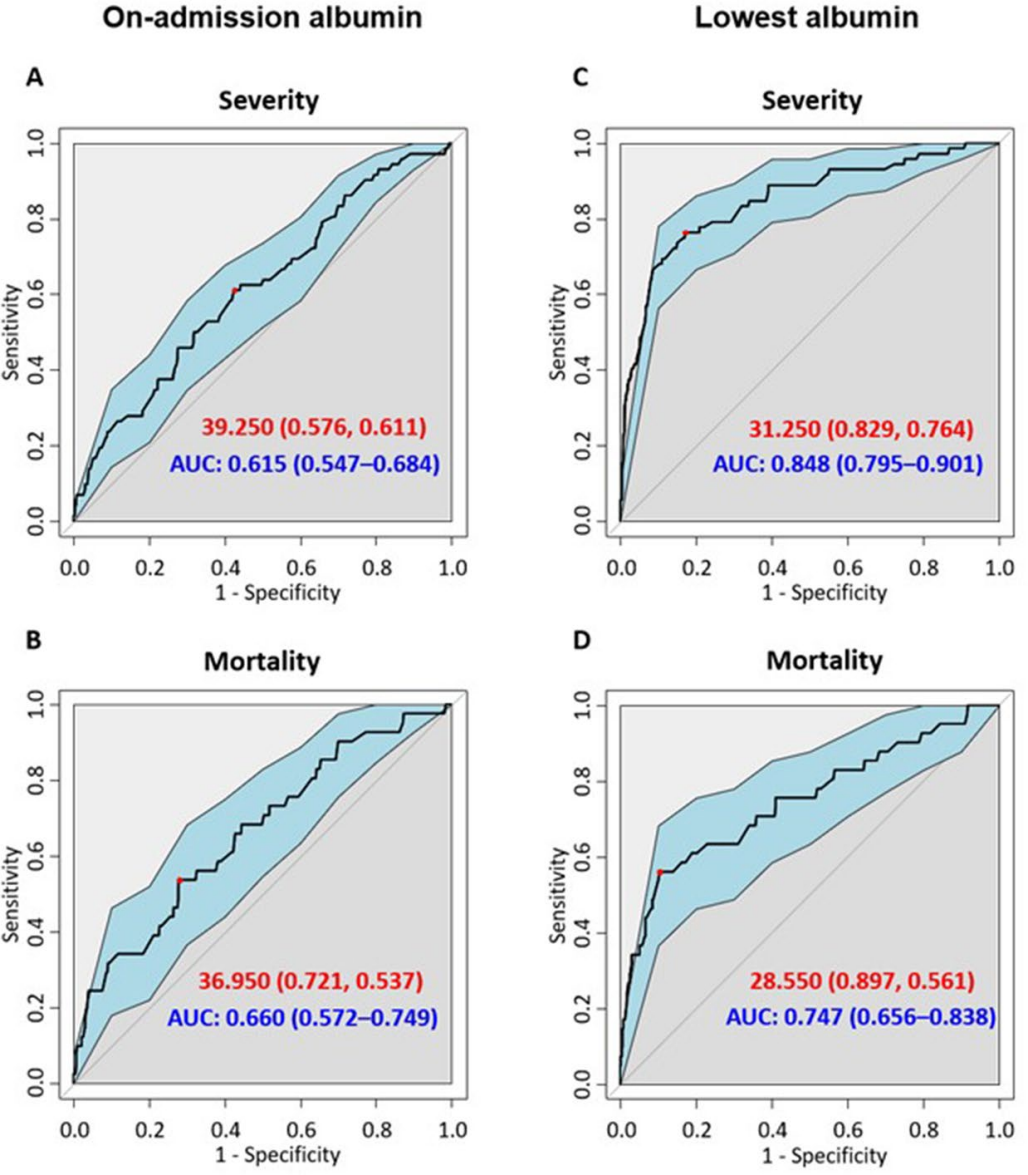
Receiver operating curves for mortality and severity. *AUC* area under the curve; best cut-offs are shown in red.

On-admission albumin levels have an AUC of 0.615 (sensitivity: 57.6%; specificity: 61.1%) for severity with a cut-off at 39.3 g/L (Fig. 4). The AUC for mortality was 0.660 (sensitivity: 72.1%; specificity: 53.7%) with a cut-off at 37.0 g/L.

These data suggest that albumin plays a crucial role in the pathophysiology and clinical outcome of AP; however, it cannot be used as a single biomarker for predicting severity and mortality. Next, we wanted to understand whether albumin loss during the course of AP is related in any way to outcome of the disease; therefore, we regrouped our patients based on the lowest measured albumin levels.

### One out of three patients suffer from hypoalbuminemia in AP during hospitalization, which dose-dependently correlates with disease severity and mortality in AP

The proportion of patients with hypoalbuminemia anytime during hospitalization was 35.7% (454 patients). A significant, dose-dependent increase was seen in the low albumin groups (Group 5–7) compared to the normal albumin group as regards the rate of all examined systemic and local complications (Figs. 1, 2). The lowest measured albumin levels throughout hospitalization (n = 1272) were significantly and dose-dependently associated with severity (p < 0.001), mortality (p < 0.001), length of stay (p < 0.001) and maximum CRP values (p < 0.001) (Fig. 3).

### Moderate and severe AP and mortality are associated with significantly lower albumin levels and greater albumin loss

Albumin loss was analysed using data from patients with at least two albumin measurements (n = 335; Sup. Fig. S18). Compared to mild cases, patients with moderate and severe AP showed a greater decrease in albumin levels (medians 5.4 vs. 9 and 15.25 g/L; p < 0.001 for both comparisons). The comparison of delta albumin between the moderate and severe groups also yielded significant results (p = 0.003). Patients who died also lost significantly more albumin during hospitalization (medians 6.7 vs. 15.75 g/L; p = 0.002). The median time to the lowest albumin levels from admission was 4 days (IQR: 3–7 days).

### AP patients with less than 25 g/L serum albumin have a 16.8-fold higher risk of death and a 48.8-fold higher risk of severe AP compared to patients with normal albumin levels

Age is an independent risk factor for severe AP and mortality, whereas hypertriglyceridemia-induced and idiopathic AP and a combination of alcoholic and biliary causes are independently associated with mortality (Table 2 and Supplementary Table S4). Hypoalbuminemia below 25–29.99 g/L (OR 2.912; CI 1.176–6.893) and below 25 g/L (OR 16.828; CI 8.323–35.129) were associated with an increased risk of mortality (Table 2). In a separate analysis, hypoalbuminemia (< 35 g/L) was also an independent risk factor for mortality (OR 4.185; CI 2.286–8.039) (Table S4). Furthermore, hypoalbuminemia anytime during hospitalization was associated with a higher risk for severe AP (OR 10.664; CI 6.188–19.614), and a gradual increase of odds ratios can be observed in the low albumin groups (OR 2.359; CI 1.030–5.240 for Group 5; OR 11.709; CI 6.038–23.515 for Group 6; and OR 48.761; CI 25.276–98.908 for Group 7).

**Table 2:** Logistic regression for severity and mortality using the lowest measured albumin cohort.

Predictor	β	SE	OR	95% CI	p
**Lowest measured albumin (n = 1272)—mortality**					
On-admission albumin level					
30–34.99 g/L (vs. ≥ 35 g/L)	− 0.016	0.531	0.984	0.313–2.621	0.976
25–29.99 g/L (vs. ≥ 35 g/L)	1.069	0.448	2.912	1.166–6.893	**0.017**
< 25 g/L (vs. ≥ 35 g/L)	2.823	0.365	16.828	8.323–35.129	** < 0.001**
Age					
Per years	0.043	0.012	1.044	1.021–1.070	** < 0.001**
Gender					
Female (vs. male)	− 0.352	0.347	0.703	0.352–1.380	0.309
Aetiology					
Alcohol (vs. biliary)	0.909	0.523	2.481	0.880–6.960	0.083
HTG (vs. biliary)	1.569	0.766	4.803	0.914–19.900	**0.041**
Biliary + alcohol (vs. biliary)	1.651	0.793	5.215	0.949–22.798	**0.037**
Biliary + HTG (vs. biliary)	− 12.335	786.272	–	–	0.987
Alcohol + HTG (vs. biliary)	1.356	0.793	3.880	0.709–17.009	0.087
Idiopathic (vs. biliary)	1.402	0.402	4.063	1.878–9.181	** < 0.001**
Other (vs. biliary)	0.213	0.807	1.237	0.182–5.045	0.792
**Lowest measured albumin (n = 1272)—severity**					
On-admission albumin					
30–34.99 g/L (v. ≥ 35 g/L)	0.858	0.410	2.359	1.030–5.240	**0.036**
25–29.99 g/L (v. ≥ 35 g/L)	2.460	0.345	11.709	6.038–23.515	** < 0.001**
< 25 g/L (v. ≥ 35 g/L)	3.887	0.346	48.761	25.276–98.908	** < 0.001**
Age					
Per years	0.032	0.009	1.032	1.015–1.051	** < 0.001**
Gender					
Female (vs. male)	− 0.332	0.274	0.718	0.417–1.225	0.226
Aetiology					
Alcohol (vs. biliary)	0.093	0.403	1.097	0.492–2.403	0.818
HTG (vs. biliary)	1.060	0.565	2.885	0.910–8.476	0.061
Biliary + alcohol (vs. biliary)	0.172	0.778	1.188	0.222–5.006	0.825
Biliary + HTG (vs. biliary)	− 13.429	753.256	–	–	0.986
Alcohol + HTG (vs. biliary)	0.497	0.657	1.643	0.422–5.688	0.450
Idiopathic (vs. biliary)	0.541	0.320	1.718	0.915–3.218	0.091
Other (vs. biliary)	0.008	0.547	1.008	0.310–2.744	0.988

*HTG* hypertriglyceridemia, *β* β coefficient, *SE* standard error, *OR* odds ratio, *CI* confidence interval.

### The lowest albumin values have good and fair predictive value for severity and mortality in acute pancreatitis

The lowest measured albumin levels have higher AUC values: 0.848 for severity and 0.747 for mortality (Fig. 3). The best cut-off values were 31.3 g/L for severity (sensitivity: 82.9%; specificity: 76.4%) and 28.6 g/L for mortality (sensitivity: 89.9%; specificity: 56.1%). The day of the lowest albumin measurement ranged from 1 to 56 days, with a median of 2 days. Most patients only had a single measurement around the time of admission.

## Discussion

To date, this is the most comprehensive evaluation of AP patients with hypoalbuminemia, using the largest, prospectively collected, high-quality dataset^8,9^.

We found that almost one-fifth of patients had hypoalbuminemia on admission (19%), and a further 25% developed hypoalbuminemia during hospitalization, meaning that every third patient was affected.

In our analysis, hypoalbuminemia under 25 g/L anytime during hospitalization was independently associated with a more than 47-fold higher chance for severe AP and a more than 16-fold higher chance for mortality.

Our findings are consistent with results for hypoalbuminemia in other diseases. Hypoalbuminemia was a prominent risk factor in community-acquired bloodstream infection with severe sepsis and septic shock^10^. A retrospective analysis of data from more than 20,000 emergency medical patients in Ireland found that hypoalbuminemia is independently associated with 30-day in-hospital mortality, with a non-linear relationship between mortality and on-admission albumin levels^11^. Moreover, in a secondary analysis of a prospective cohort, AP patients with multiorgan failure (MOF; n = 18) demonstrated a sharper decline in serum albumin (P < 0.001) compared to non-MOF patients (n = 39)^12^.

We have not only proved that hypoalbuminemia is a risk factor, but have also shown the dose-dependent relation between low albumin levels and severity, mortality, number of patients with any local complications, number of patients developing organ failure and maximum CRP levels in both analyses (on-admission and lowest measured albumin levels).

These relations can be explained by the numerous physiological functions of human serum albumin. Albumin was long considered a negative acute-phase protein, with decreasing production giving way to inflammatory cytokines in inflammation^13^. Serum albumin levels undoubtedly decrease in inflammatory states, which may be due to a shorter half-life and a larger interstitial pool, which causes the dilution of albumin^14–16^. Capillary leak resulting from inflammatory processes plays a role in the decline of serum albumin, but it is argued that the escape of albumin to the tissues may be beneficial because of its antioxidant and scavenging activity^17^. Although a more than twofold higher production rate was observed in critically ill ICU patients, this increased production is still not able to balance the higher demand. This can be considered as a relative synthetic insufficiency of hepatic function^18^.

Albumin loss was significantly associated with severity and mortality in our analysis. However, only 51.7% of patients in the HPSG database had albumin measurements at least once during their hospitalization, and 13.6% had them at least twice during that time. This highlights how neglected albumin measurements are in AP.

On admission albumin levels were found to have poor predictive values for mortality and severity. Previous studies were mainly retrospective and had a much smaller sample size^5,19,20^. They only assessed the predictive value of serum albumin for persistent organ failure and peripancreatic infection, or were limited to severe AP.

From the clinician’s point of view, the decline of serum albumin levels—regardless of on-admission albumin levels—signals clinical worsening and may aid in identifying high-risk AP patients. However, clinicians mostly miss the opportunity to pre-emptively and frequently measure serum albumin, thus delaying timely intervention.

To date, no clinical trial examined therapeutic albumin administration in AP. As we know, albumin is similarly associated with outcomes in sepsis and septic shock; randomized controlled trials in this field could be a start^17,21^. The controversial results of studies and meta-analyses in this field may be explained by heterogeneous patient populations and the time sensitivity of this treatment^22^.

To further exploit the potential in therapeutic albumin administration in AP, more detailed clinical studies are needed to identify the patient subpopulations benefiting the most from this therapeutic option.

### Strengths and limitations

We conducted the most extensive, most comprehensive cohort study on the role of hypoalbuminemia in acute pancreatitis to date. We analysed high-quality data from a prospective, international, multicentric registry. We identified hypoalbuminemia as an independent risk factor in AP, present in at least every third patient. We also found a dose-dependent relationship between albumin levels and main outcomes, which was previously not described.

Among the limitations, we must mention the arbitrary classification of albumin levels (except for the low-normal cut-off), the missing data on albumin levels and albumin administration during hospital stay, and the limited number of albumin measurements during the hospital stay, which could introduce bias. The limited number of albumin measurements did not enable more detailed analyses of serum albumin at different time points. Our analysed cohorts differed from the total cohort in some aspects, thus potentially signalling performance bias, as albumin measurements are more frequently ordered for patients with expected hypoalbuminemia.

## Conclusion

Hypoalbuminemia is remarkably common in AP (seen in 19% of patients on admission and 35.7% during hospitalization) and represents an independent risk factor for severity and mortality. Importantly, albumin loss during hospitalization was also associated with severity and mortality, suggesting that routine monitoring of serum albumin is recommended and that albumin administration should be examined as a therapeutic intervention in AP.

### Implications for research

Clinical trials are needed to assess the potential benefit of albumin replacement in AP.

### Implications for practice

(1) Albumin levels should be measured for all AP patients, (2) albumin levels should be controlled at least in those patients whose condition is worsening during AP, and (3) albumin administration should at least be considered in patients with severe hypoalbuminemia (< 25 g/L).

## Methods

### Study design and definitions

This analysis of an international, prospective, multicentre cohort was conducted using data from the Acute Pancreatitis Registry operated by the Hungarian Pancreatic Study Group (HPSG)^23^. Patient data were collected from establishment of the registry to 31 December 2019 on electronic case report forms and validated using a four-tiered data validation protocol. Contributing centres are shown in the supplementary material (Table S1 and Fig. S1). The registry was approved by the Scientific and Research Ethics Committee of the Medical Research Council of Hungary (222254-1/2012/EKU) in 2012. It conforms to the Declaration of Helsinki, as revised in 2013. All participants provided written informed consent. Data collection and validation are detailed by Párniczky et al.^24^. The Hungarian Pancreatic Study Group published analyses from the registry, the population of which may overlap with our analysed cohort^24–34^.

Diagnosis of AP was established using the IAP/APA guidelines^35^, while severity and complications were defined using the Revised Atlanta Classification^1^.

### Participants

Analyses were performed on patients’ data with albumin measurement anytime during hospitalization (lowest measured albumin cohort, n = 1272) and in the first 48 h of hospitalization (on-admission albumin cohort, n = 1149) to answer a post-hoc clinical research question. The cut-off value between the low and normal albumin group was 35 g/L in both cases, based on the commonly used lower normal value. Subjects were further divided into seven subgroups (Groups 1 to 7) using the lowest (n = 1272) or first measured (n = 1149) albumin values.

The analyses of albumin change involved selected patients (n = 335) with at least two albumin measurements. Delta albumin was calculated as the difference between the first and lowest measured albumin levels.

### Statistical analysis

Descriptive statistics are presented as the median with 25% and 75% percentiles (IQR) or mean with standard deviation (SD) for continuous variables and as numbers and proportions for categorical variables.

The Chi-squared test or Fisher’s exact test was used to assess the relationship between categorical variables. The Mann–Whitney U test or Kruskal–Wallis test followed by Dunnett’s post hoc test was used to evaluate differences between groups in the case of continuous variables.

Multivariate binary logistic regression analysis was performed to identify the risk factors independently associated with severe disease and mortality. Odds ratios (OR) with 95% confidence intervals (CI) were calculated.

The Receiver Operator Characteristic (ROC) curve and Area Under the Curve (AUC) with 95% CI were used to identify the ability of albumin levels to predict the mortality or severity of AP (The various AUC values were classified as follows: between 0.5 and 0.6—fail; between 0.6 and 0.7—poor; between 0.7 and 0.8—fair; between 0.8 and 0.9—good; and over 0.9—excellent.) Best cut-offs were calculated using the Youden index^36^.

P < 0.05 was considered statistically significant, except for the Kruskal–Wallis test followed by Dunnett’s post hoc test, where p < 0.025 was considered statistically significant.

All analyses were carried out in R statistical software, version 4.0.2 (R Core Team, 2020, Vienna, Austria), packages: pROC (v. 1.17.0.1) and PMCMRplus (v. 1.9.0.)^37,38^.

### Representativity

The main characteristics of the analysed cohorts are consistent with the literature data. However, they differed significantly from the entire cohort (n = 2461) in terms of severity, length of stay and mortality (Fig. S2).

### Reporting

We report our results following The Strengthening the Reporting of Observational Studies in Epidemiology (STROBE) Statement, using the checklist provided^39^.

## Supplementary Information

Supplementary Information.

## Data Availability

The full dataset is available upon reasonable request.
