# Supplementary material for: Hypoalbuminemia affects one third of acute pancreatitis patients and is independently associated with severity and mortality
Source: Sci Rep. 2021 Dec 17;11:24158. doi: 10.1038/s41598-021-03449-8 (PMC8683470; doi:10.1038/s41598-021-03449-8)
Supplement: Supplementary file 1 — Supplementary Information. [file 41598_2021_3449_MOESM1_ESM.docx]

**Supplementary material**

**Hypoalbuminemia affects one third of acute pancreatitis patients and is independently associated with severity and mortality**

Klementina Ocskay^1,2^, Zsófia Vinkó^1^, Dávid Németh^1^, László Szabó^1,3^, Judit Bajor^4^, Szilárd Gódi^4^, Patrícia Sarlós^4^, László Czakó^5^, Ferenc Izbéki^6^, József Hamvas^7^, Mária Papp^8^, Márta Varga^9^, Imola Török^10^, Artautas Mickevicius^11,12^, Ville Sallinen^13^, Elena Ramirez Maldonado^14^, Shamil Galeev^15^, Alexandra Mikó^1^, Bálint Erőss^1,2,16^, Marcell Imrei^1,2^, Péter Jenő Hegyi^1,2,16^, Nándor Faluhelyi^17^, Orsolya Farkas^17^, Péter Kanizsai^18^, Attila Miseta^19^, Tamás Nagy^19^, Roland Hágendorn^20^, Zsolt Márton^20^, Zsolt Szakács^1,20^, Andrea Szentesi^1,2,21^, Péter Hegyi^1,2,16#^ and Andrea Párniczky^1,2,22#^

^1^Institute for Translational Medicine, Szentágothai Research Centre, Medical School, University of Pécs, Pécs, Hungary

^2^Centre for Translational Medicine, Semmelweis University, Budapest, Hungary

^3^ Centre for Translational Medicine, Department of Medicine, University of Szeged, Szeged, Hungary

^4^Division of Gastroenterology, First Department of Medicine, Medical School, University of Pécs, Pécs, Hungary

^5^Department of Medicine, University of Szeged, Szeged, Hungary

^6^Szent György University Teaching Hospital of Fejér County, Székesfehérvár, Hungary

^7^Peterfy Hospital, Budapest, Hungary

^8^Department of Internal Medicine, Division of Gastroenterology, University of Debrecen, Debrecen, Hungary

^9^Dr. Réthy Pál Hospital, Békéscsaba, Hungary

^10^County Emergency Clinical Hospital – Gastroenterology and University of Medicine, Pharmacy, Sciences and Technology, Targu Mures, Romania

^11^Vilnius University Hospital Santaros Clinics, Vilnius, Lithuania

^12^Clinics of Abdominal Surgery, Nephrourology and Gastroenterology, Faculty of Medicine, Vilnius University, Vilnius, Lithuania

^13^Department of Transplantation and Liver Surgery, Helsinki University Hospital and University of Helsinki, Helsinki, Finland

^14^General Surgery, Consorci Sanitari del Garraf, Sant Pere de Ribes, Barcelona, Spain

^15^North-Western State Medical University, Saint-Petersburg, Russia

^16^Division of Pancreatic Diseases, Heart and Vascular Center, Semmelweis University, Budapest, Hungary

^17^Department of Medical Imaging, Medical School, University of Pécs, Pécs, Hungary

^18^Department of Emergency Medicine, Medical School, University of Pécs, Pécs, Hungary

^19^Department of Laboratory Medicine, Medical School, University of Pécs, Pécs, Hungary

^20^First Department of Medicine, Medical School, University of Pécs, Pécs, Hungary

^21^Centre for Translational Medicine, Department of Medicine, University of Szeged, Szeged, Hungary

^22^Heim Pál National Paediatric Institute, Budapest, Hungary

#Contributed equally as last authors

Correspondence to:

Andrea Párniczky, MD, PhD

Institute for Translational Medicine, Medical School, University of Pécs

H-7624 Pécs, Szigeti u. 12., Hungary

[andrea.parniczky@gmail.com](mailto:andrea.parniczky@gmail.com); +(36-30) 290-1728

[Table S1 - List of centers 4](#_Toc85441857)

[Figure S1 – Map of centers 5](#_Toc85441858)

[Figure S2 – Representativity of the analysed cohorts 6](#_Toc85441859)

[Figure S3 – Baseline characteristics of the on-admission albumin cohort 7](#_Toc85441860)

[Figure S4 – Personal medical history 8](#_Toc85441861)

[Figure S5 – Signs and symptoms 9](#_Toc85441862)

[Table S2 – Fulfillment of diagnostic criteria 10](#_Toc85441863)

[Figure S6 – Pancreatic enzyme levels and inflammatory markers without outliers 11](#_Toc85441864)

[Figure S7 – Pancreatic enzyme levels and inflammatory markers with outliers 12](#_Toc85441865)

[Figure S8 – Laboratory parameters indicating kidney function without outliers 13](#_Toc85441866)

[Figure S9 – Laboratory parameters indicating kidney function with outliers 14](#_Toc85441867)

[Figure S10 – Laboratory parameters indicating liver function without outliers I. 15](#_Toc85441868)

[Figure S11 – Laboratory parameters indicating liver function with outliers I. 16](#_Toc85441869)

[Figure S12 – Laboratory parameters indicating liver function without outliers II. 17](#_Toc85441870)

[Figure S13 – Laboratory parameters indicating liver function with outliers II. 18](#_Toc85441871)

[Figure S14 – Haematological parameters and lipid levels without outliers 19](#_Toc85441872)

[Figure S15 – Haematological parameters and lipid levels with outliers 20](#_Toc85441873)

[Figure S16 – Ions and glucose levels without outliers 21](#_Toc85441874)

[Figure S17 – Ions and glucose levels with outliers 22](#_Toc85441875)

[Table S3 – Logistic regression for on-admission albumin cohort 23](#_Toc85441876)

[Figure S18 – Albumin loss 24](#_Toc85441877)

[Table S4 – Logistic regression for lowest measured albumin cohort 25](#_Toc85441878)

[Table S5 – Data quality for assessed variables 27](#_Toc85441879)

[Table S6 – Results of statistical comparisons presented on figures 29](#_Toc85441880)

| **Country** | **Center** | **n** |
| --- | --- | --- |
| Hungary | First Department of Medicine, Medical School, University of Pécs, Pécs | 609 |
|  | Szent György University Teaching Hospital of Fejér County, Székesfehérvár | 211 |
|  | First Department of Medicine, University of Szeged, Szeged | 148 |
|  | Department of Internal Medicine, University of Debrecen, Debrecen | 146 |
|  | Bajcsy-Zsilinszky Hospital and Clinic, Budapest, Hungary | 12 |
|  | Department of Emergency Medicine, University of Szeged, Szeged | 10 |
|  | Second Department of Medicine, University of Szeged, Szeged | 6 |
|  | Department of Surgery, University of Debrecen, Debrecen | 6 |
|  | Markusovszky University Teaching Hospital, Szombathely | 6 |
|  | Pándy Kálmán Hospital of Békés County, Gyula | 4 |
|  | Dr. Bugyi István Hospital, Szentes | 4 |
|  | Csongrád County Health Center, Makó | 4 |
|  | Dr. Réthy Pál Hospital, Békéscsaba | 2 |
|  | Buda Hospital of the Hospitaller Order of Saint John of God, Budapest | 1 |
|  | Heim Pál National Pediatric Institute, Budapest | 1 |
|  | Second Department of Internal Medicine, Semmelweis University, Budapest | 1 |
| Finland | Department of Transplantation and Liver Surgery, Helsinki University Hospital and University of Helsinki, Helsinki | 25 |
| Turkey | Hospital of Bezmialem Vakif University, School of Medicine, Istanbul | 20 |
| Russia | Saint Luke Clinical Hospital, St. Petersburg | 19 |
| Spain | General Surgery, Consorci Sanitari del Garraf, Sant Pere de Ribes, Barcelona | 14 |
| Croatia | Clinical Hospital Center Rijeka, Rijeka | 11 |
| Lithuania | Vilnius University Hospital, Vilnius, Lithuania | 5 |
| Romania | County Emergency Clinical Hospital of Targu Mures Hospital, University of Medicine, Pharmacy, Sciences and Technology of Targu Mures, Targu Mures | 2 |
|  | Central Military Emergency Hospital "Dr Carol Davila", Bucharest | 1 |
| Latvia | Gastroenterology, Hepatology and Nutritional Centre, Pauls Stradins Clinical University Hospital, Riga | 2 |
| Japan | Keio University, Tokyo | 1 |
| Ukraine | Bogomolets National Medical University, Kiev | 1 |
| **Total** |  | **1272** |

# Table S1 - List of centers

n: patient number
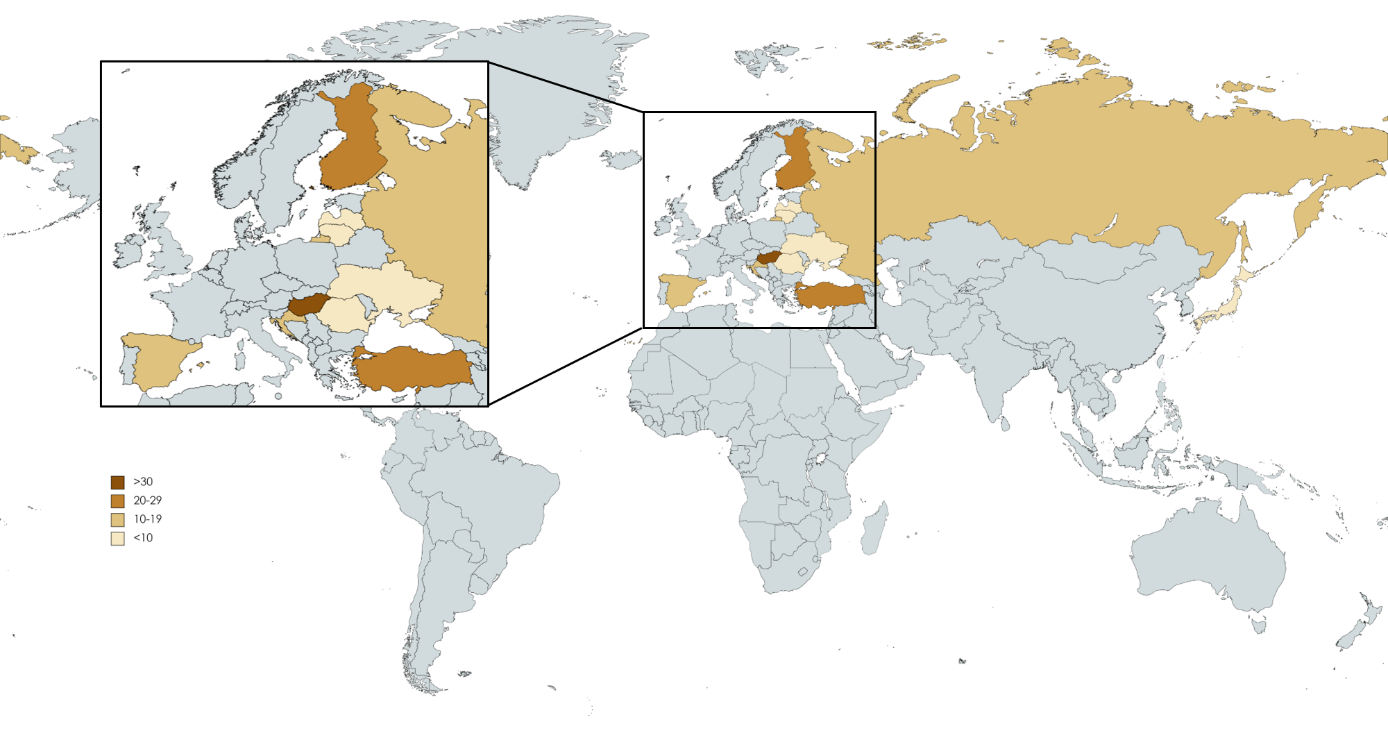


# Figure S1 – Map of centers

Created by MapChart (<https://mapchart.net/world.html>), a freely available custom map creating service.


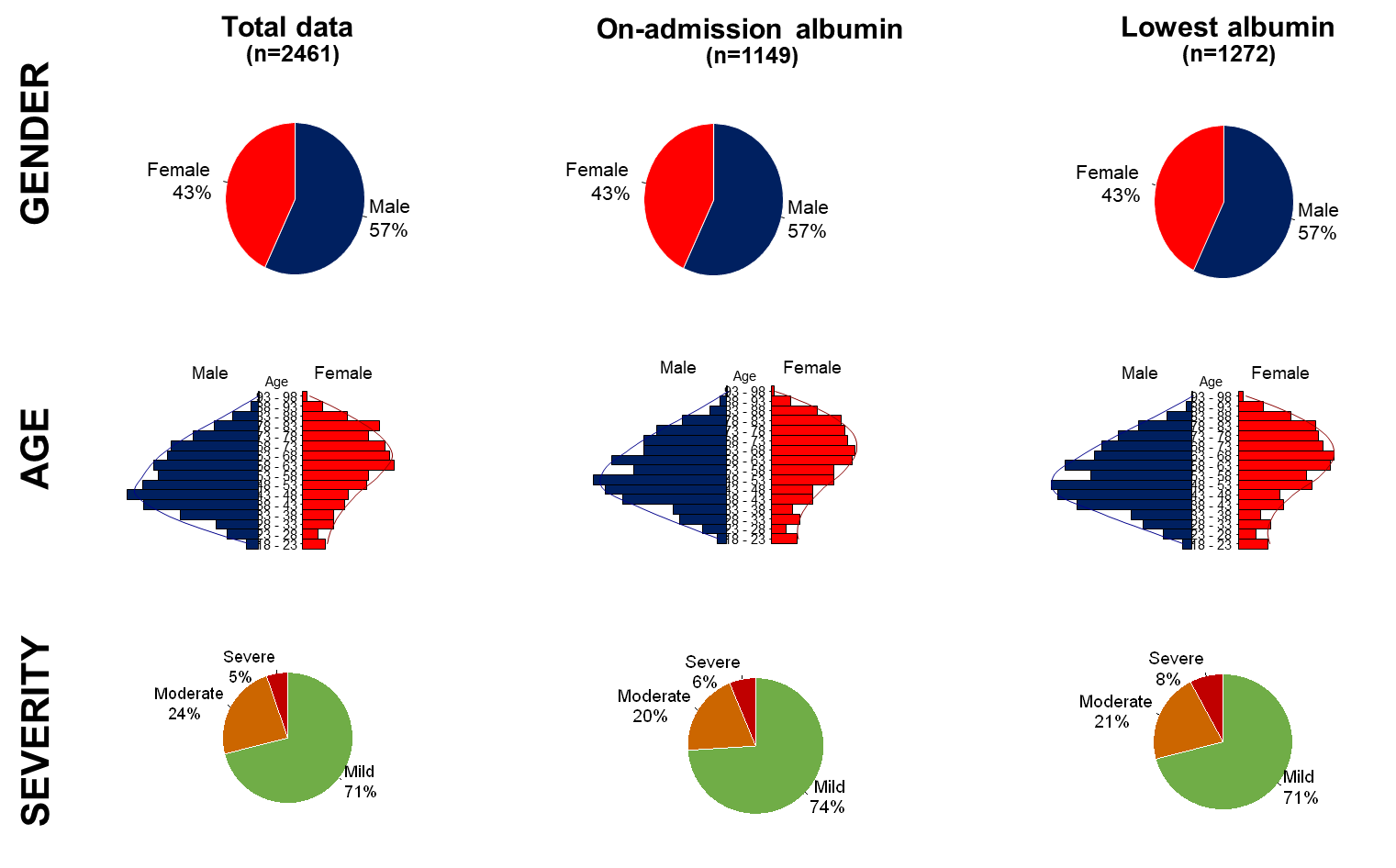


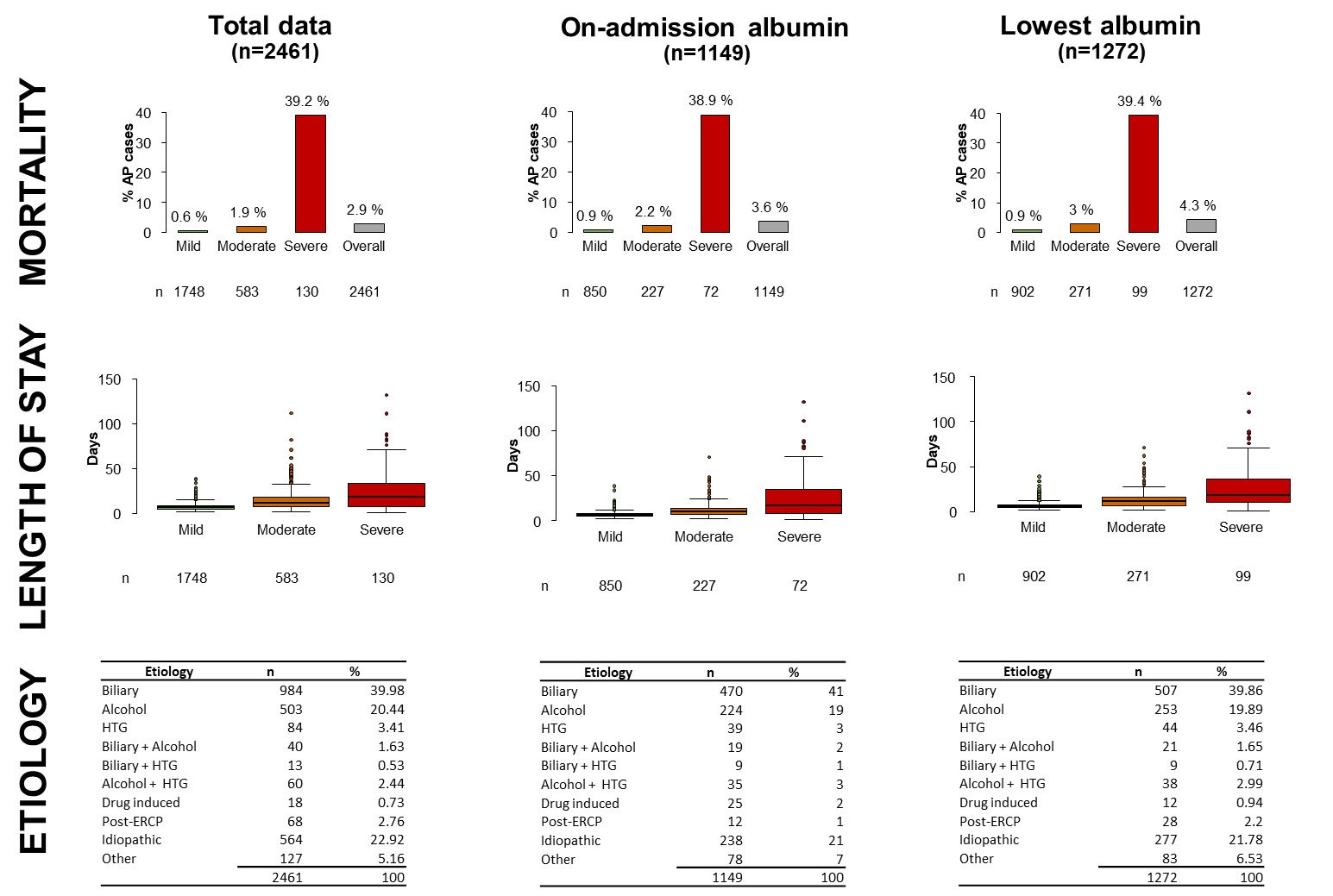


# Figure S2 – Representativity of the analysed cohorts

Cohorts were compared to the original (n=2461). The analysed cohorts differed significantly in severity (p=0.025 for on-admission and p=0.005 for lowest albumin) and length of stay (p<0.001 for both cohorts). The lowest albumin cohort also differed in mortality (p=0.026).


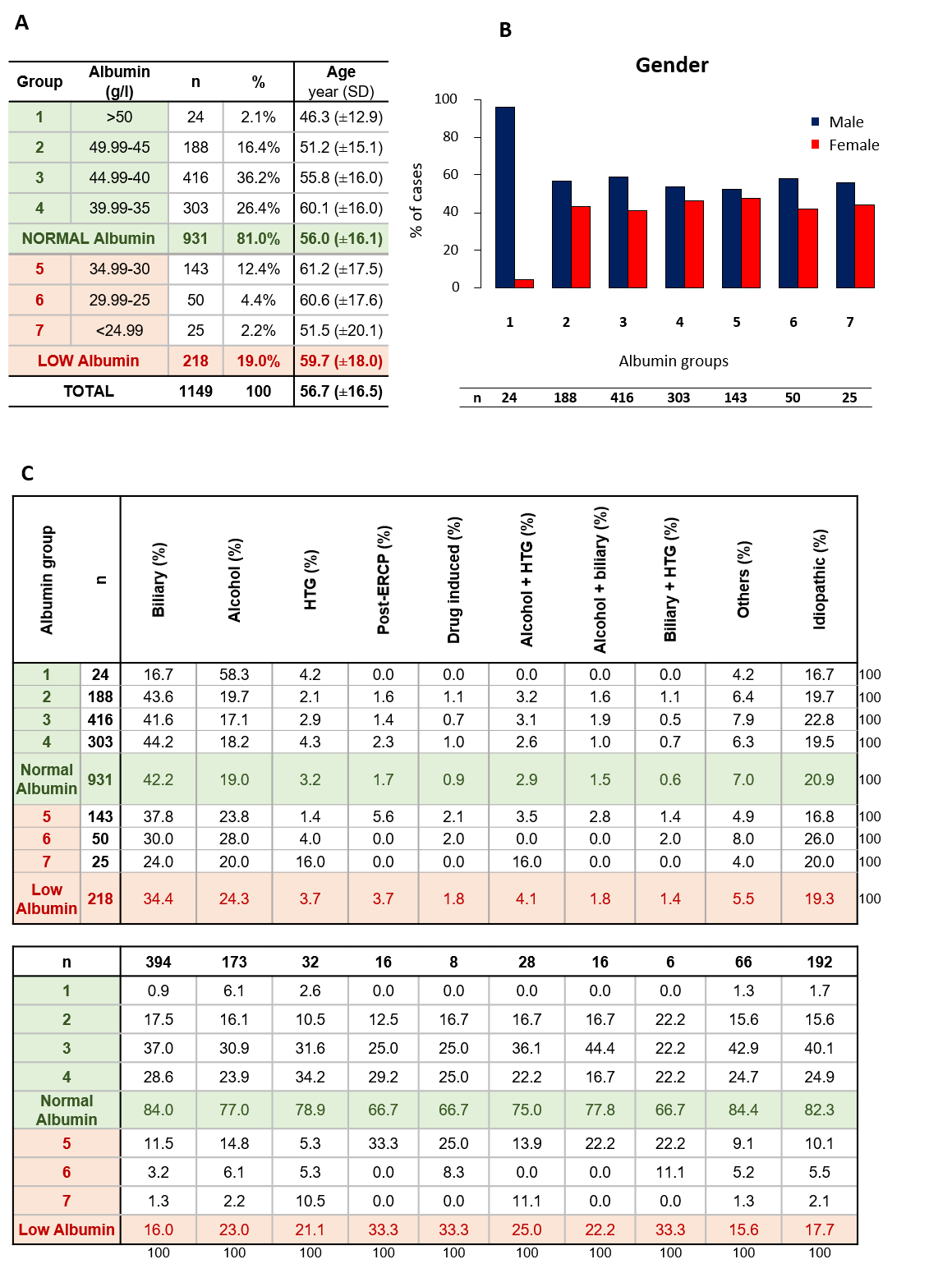


# Figure S3 – Baseline characteristics of the on-admission albumin cohort

**
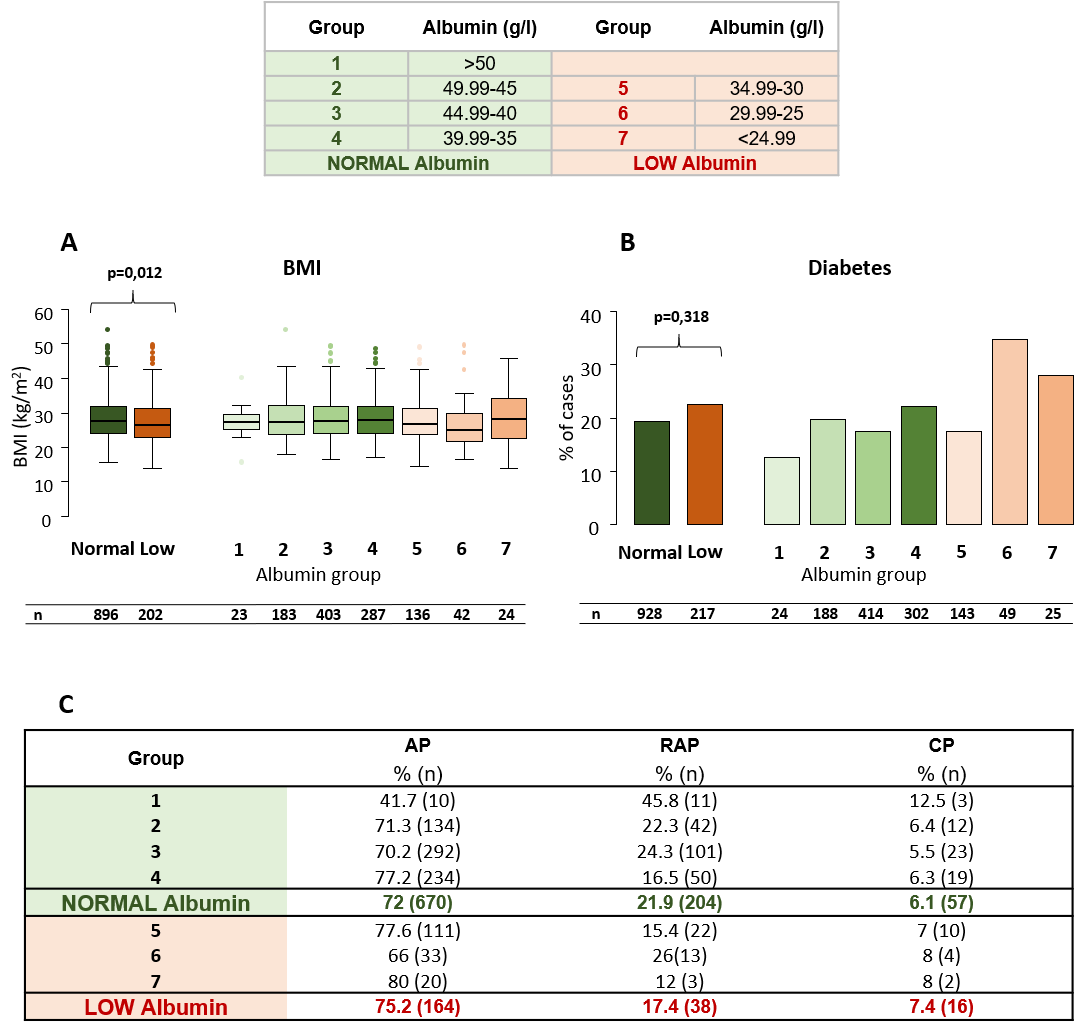
**

# Figure S4 – Personal medical history

**
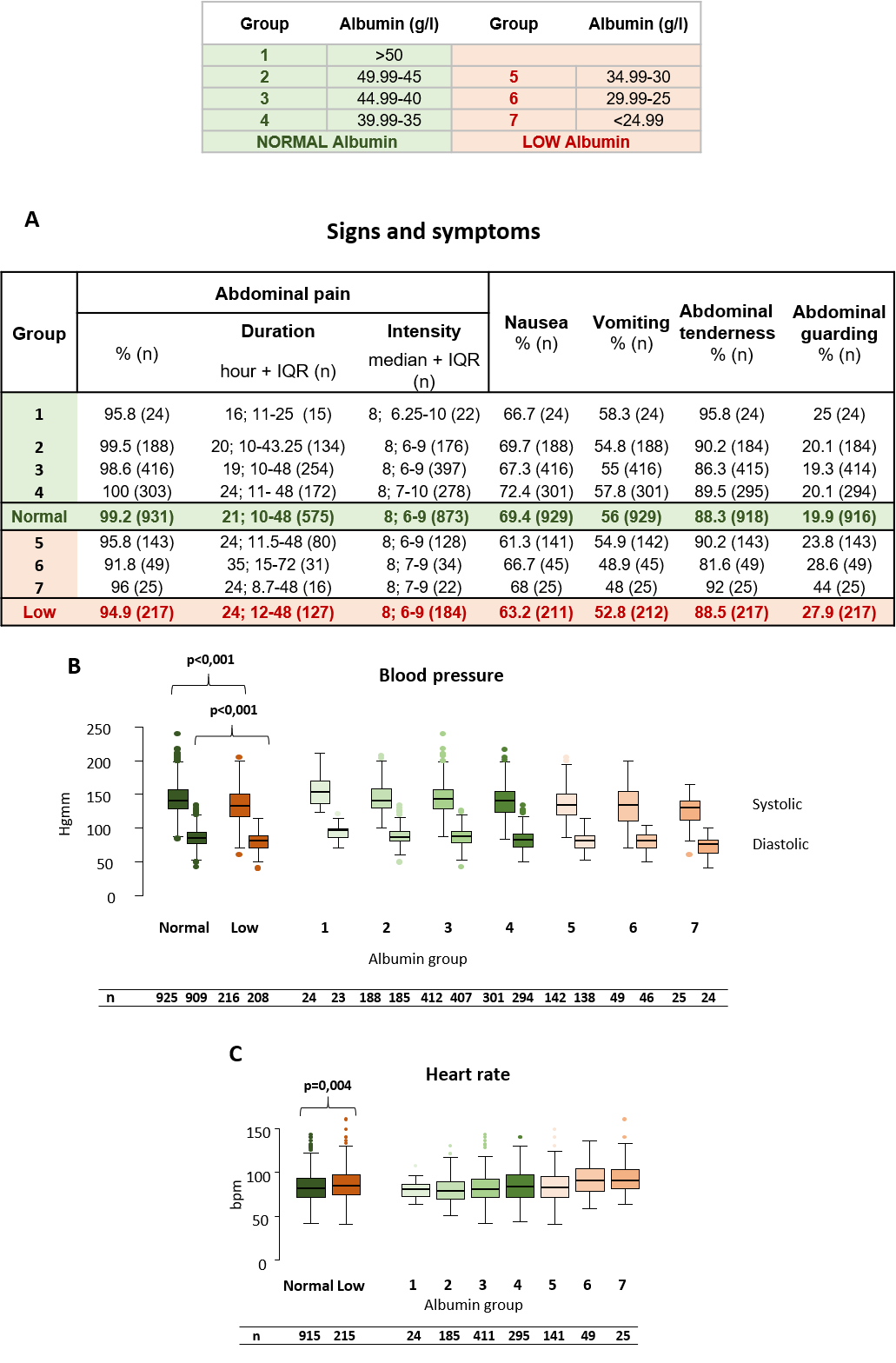
**

# Figure S5 – Signs and symptoms

**
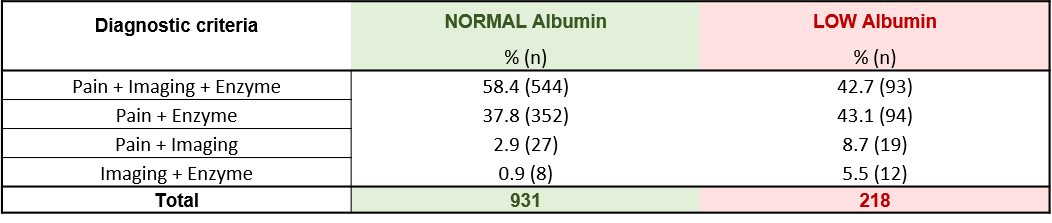
**

# Table S2 – Fulfillment of diagnostic criteria


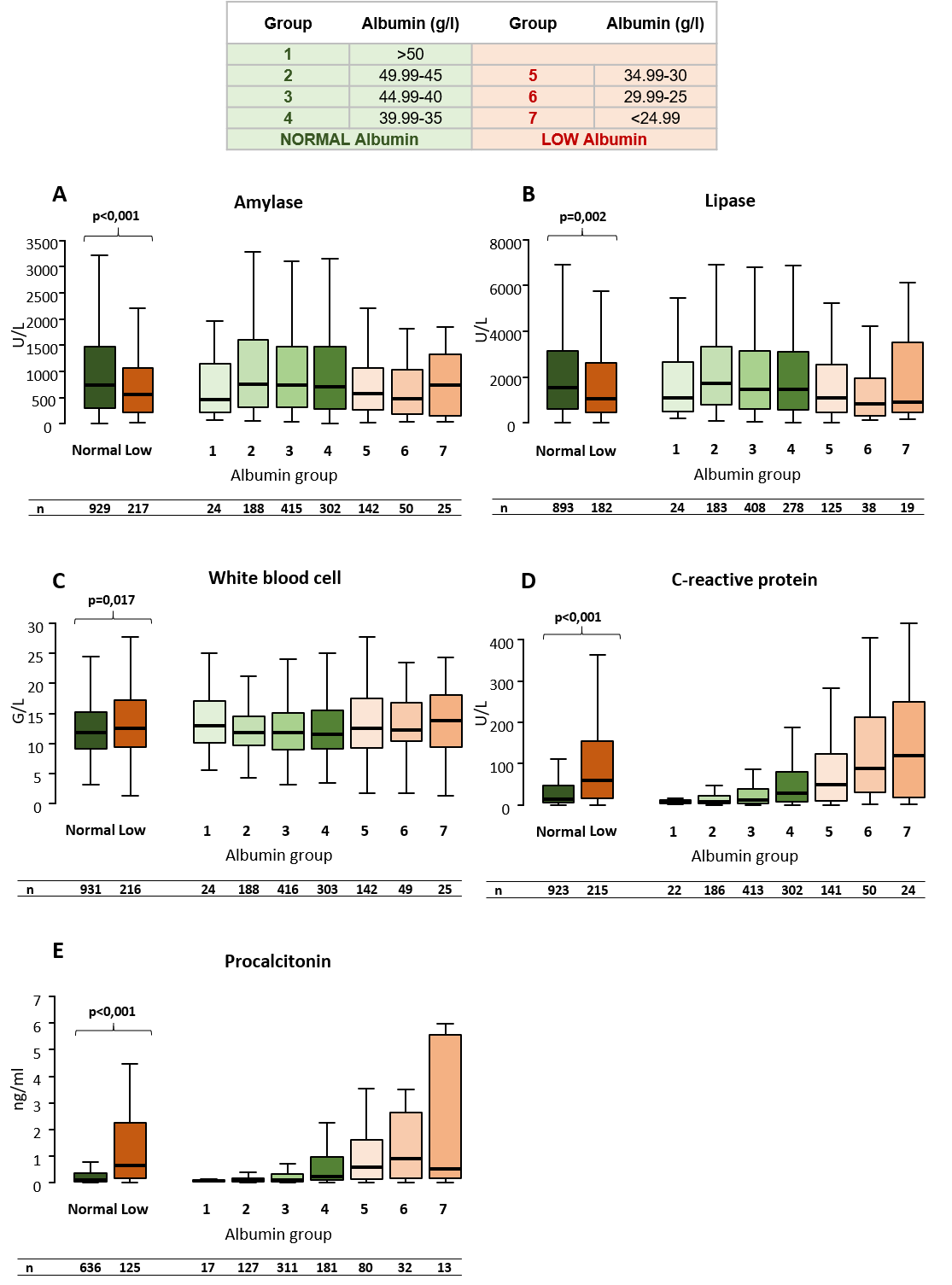


# Figure S6 – Pancreatic enzyme levels and inflammatory markers without outliers


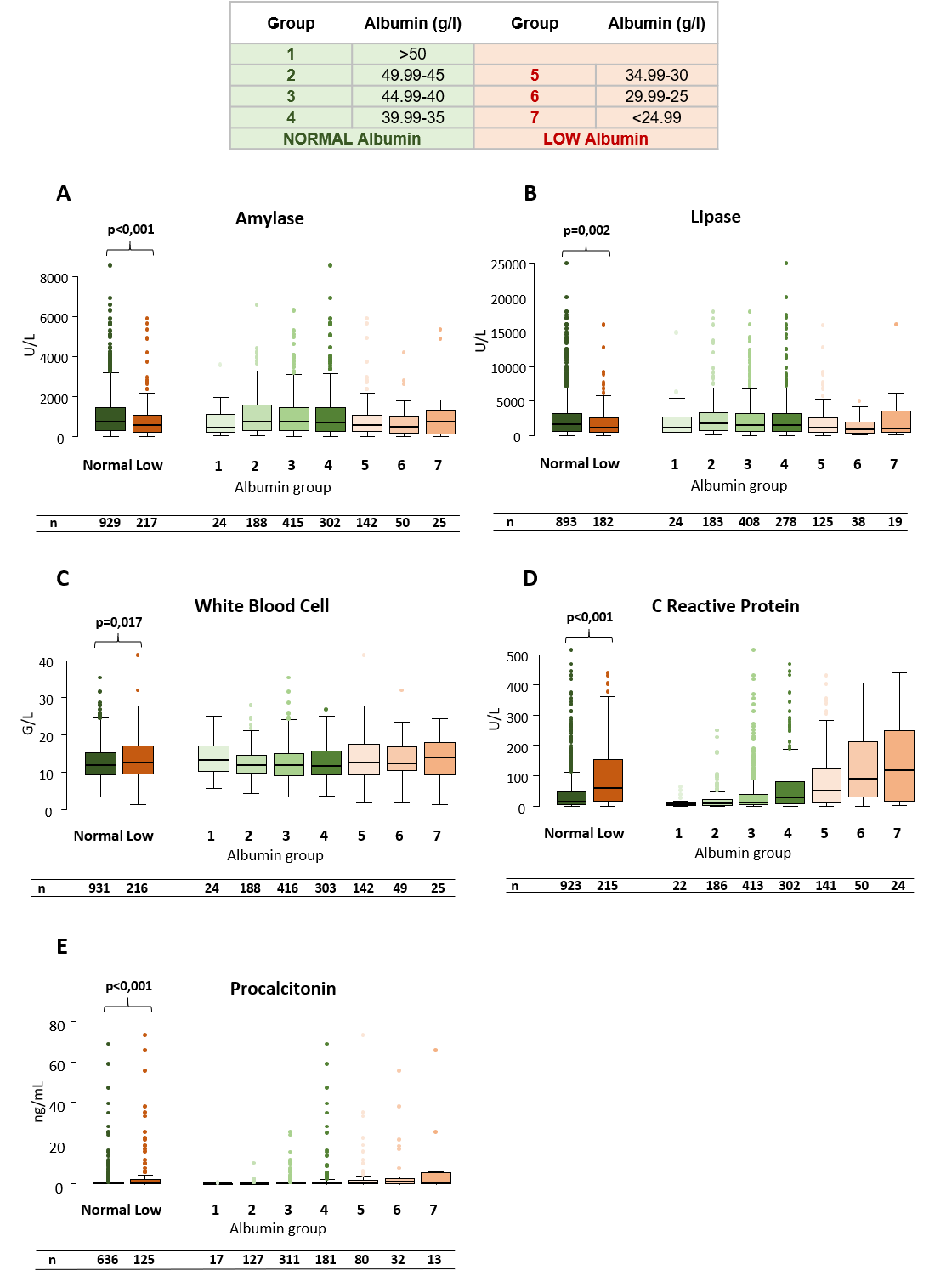


# Figure S7 – Pancreatic enzyme levels and inflammatory markers with outliers


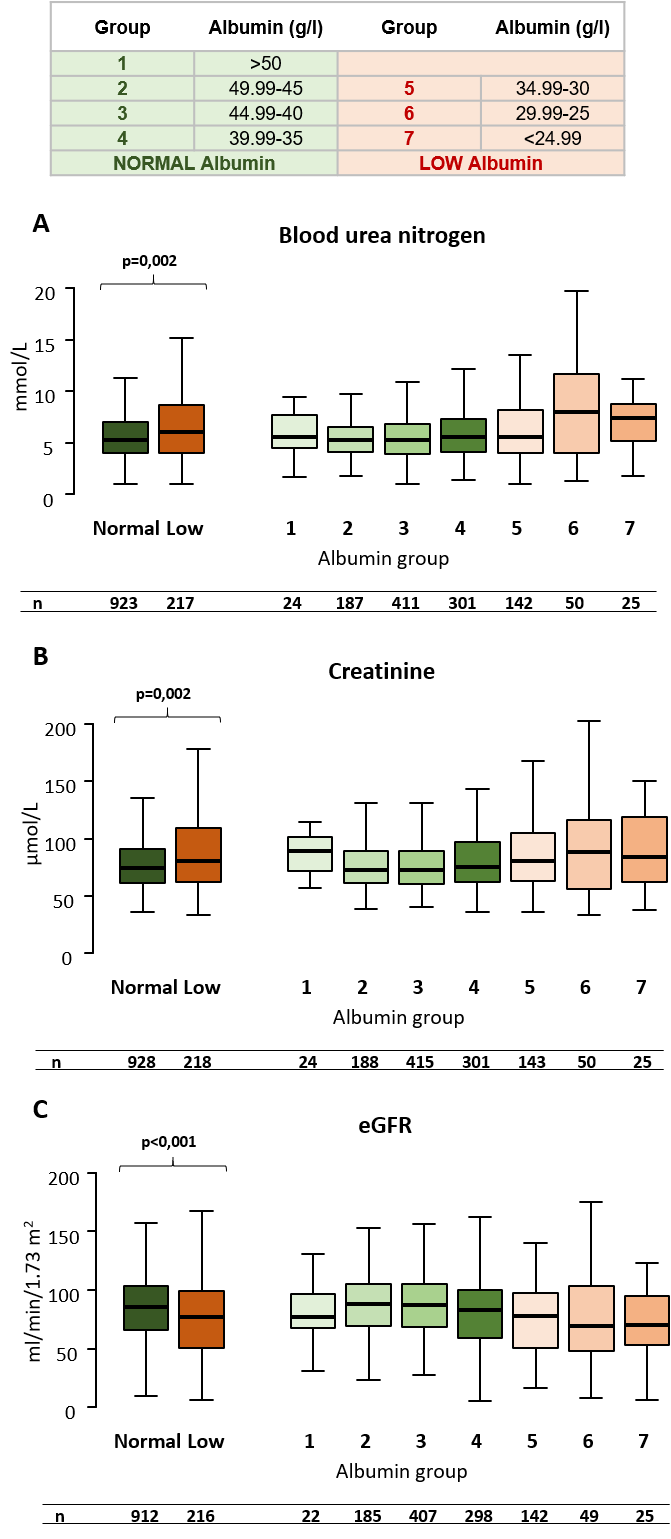


# Figure S8 – Laboratory parameters indicating kidney function without outliers


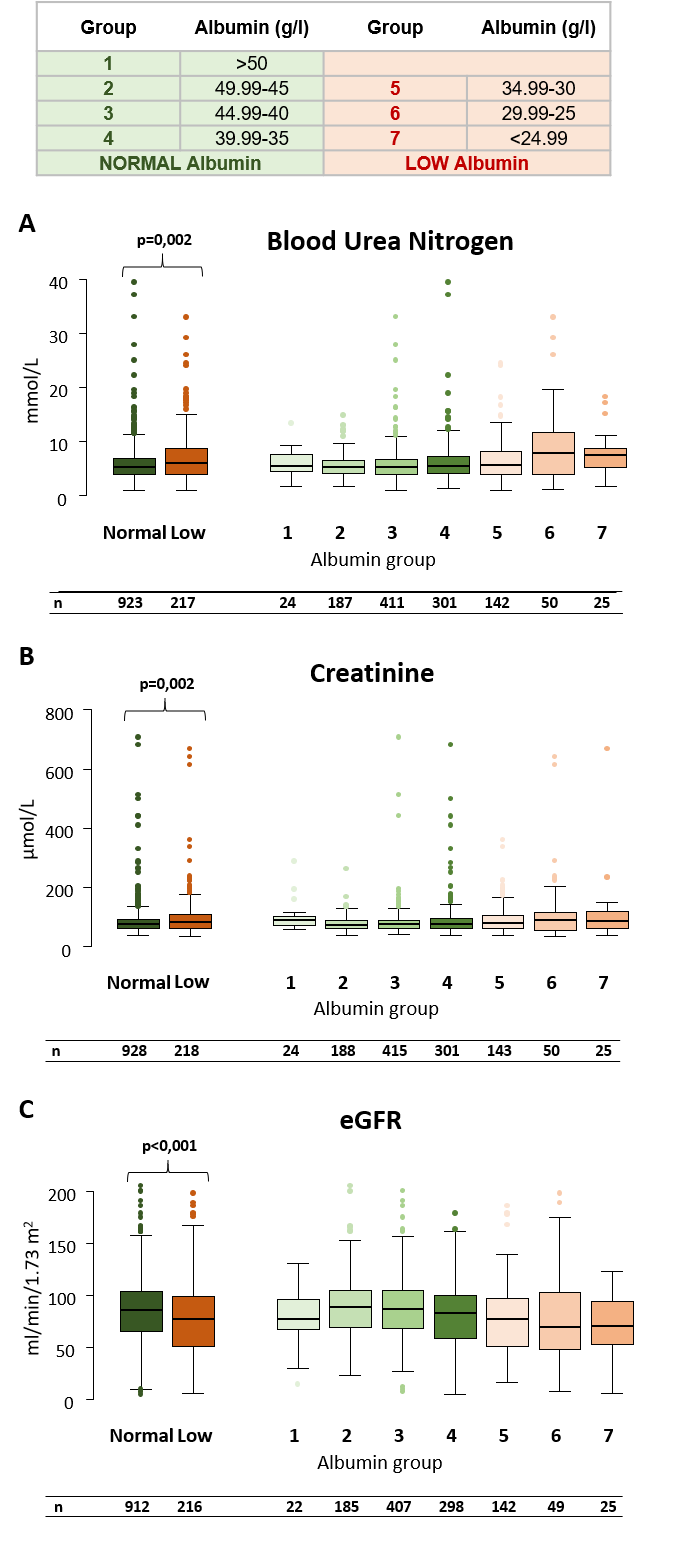


# Figure S9 – Laboratory parameters indicating kidney function with outliers


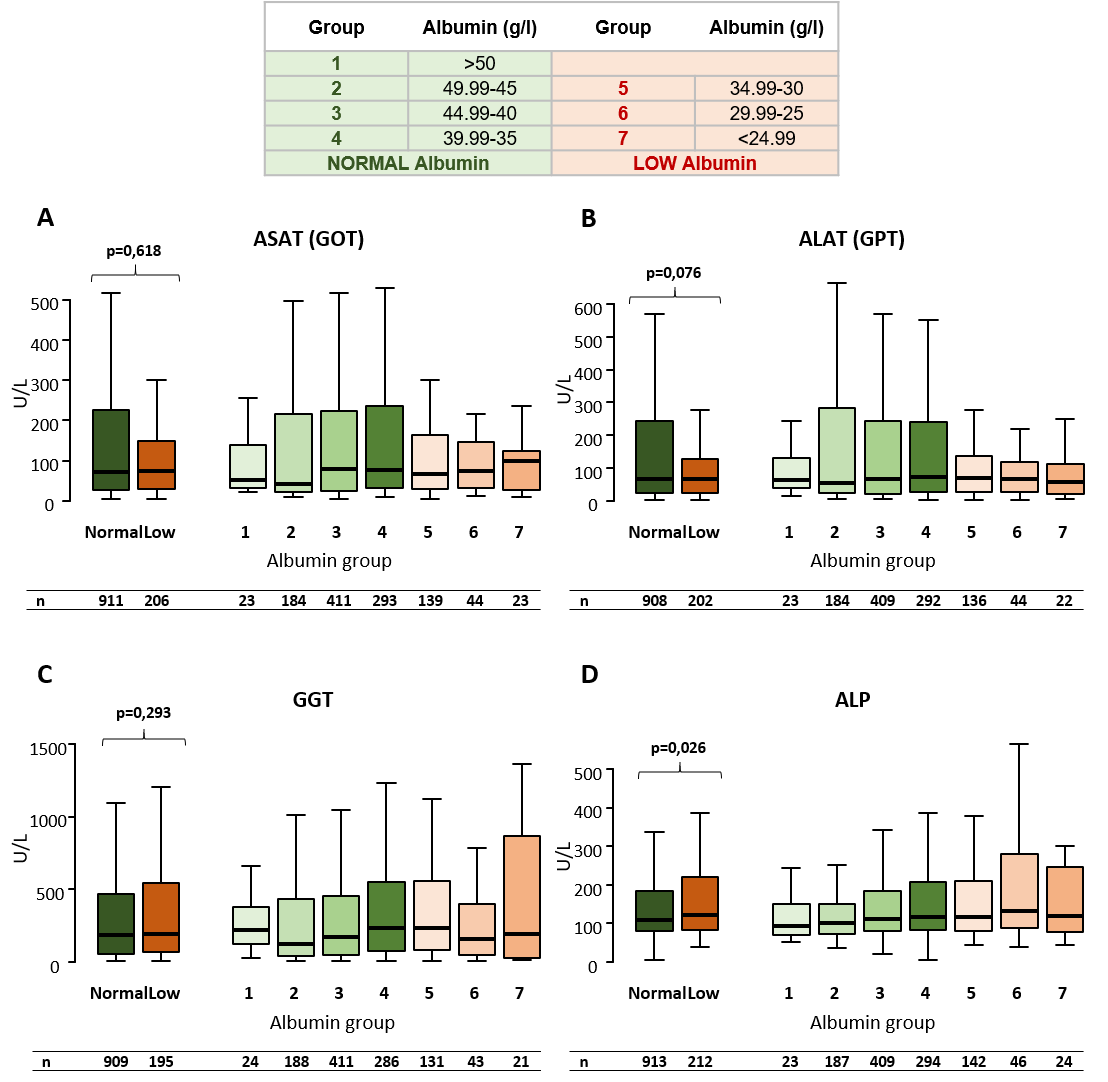


# Figure S10 – Laboratory parameters indicating liver function without outliers I.


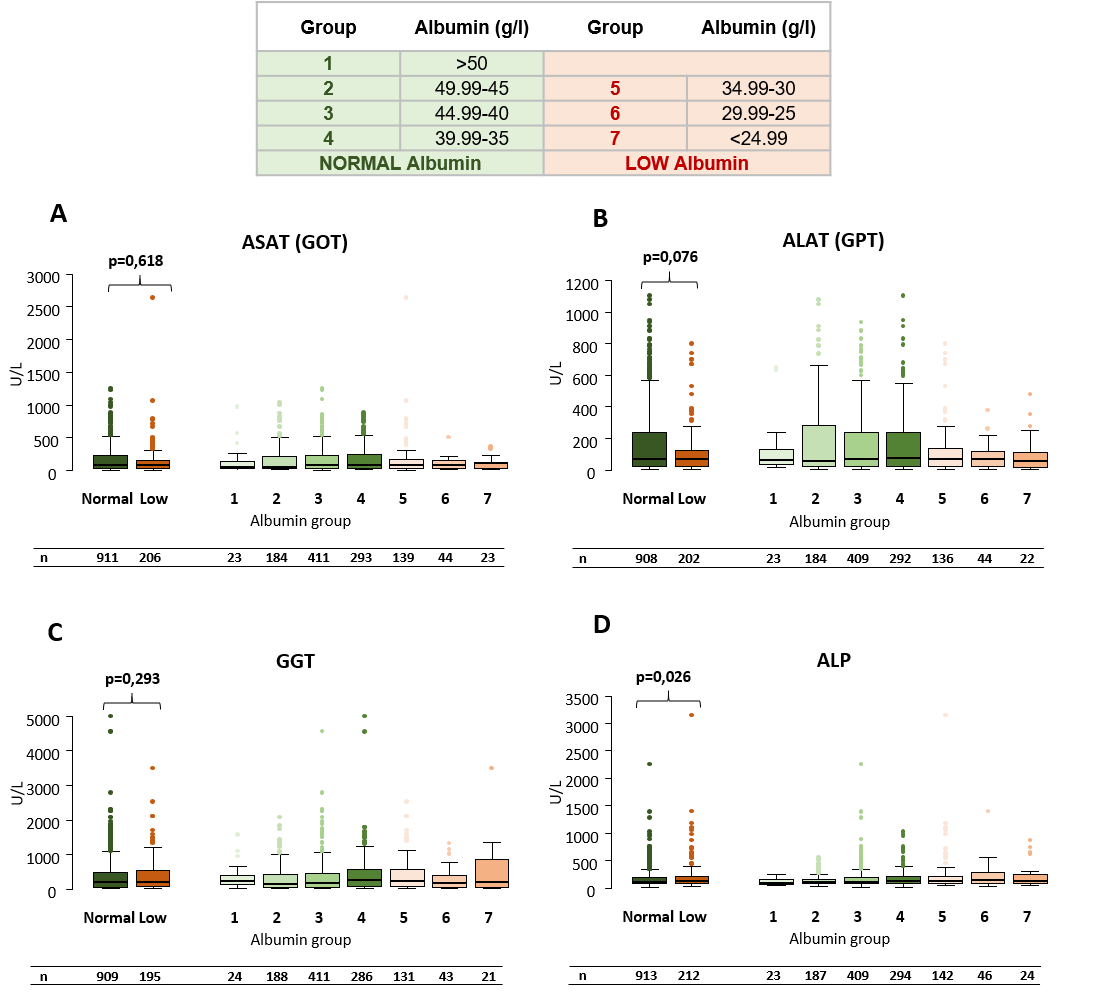


# Figure S11 – Laboratory parameters indicating liver function with outliers I.


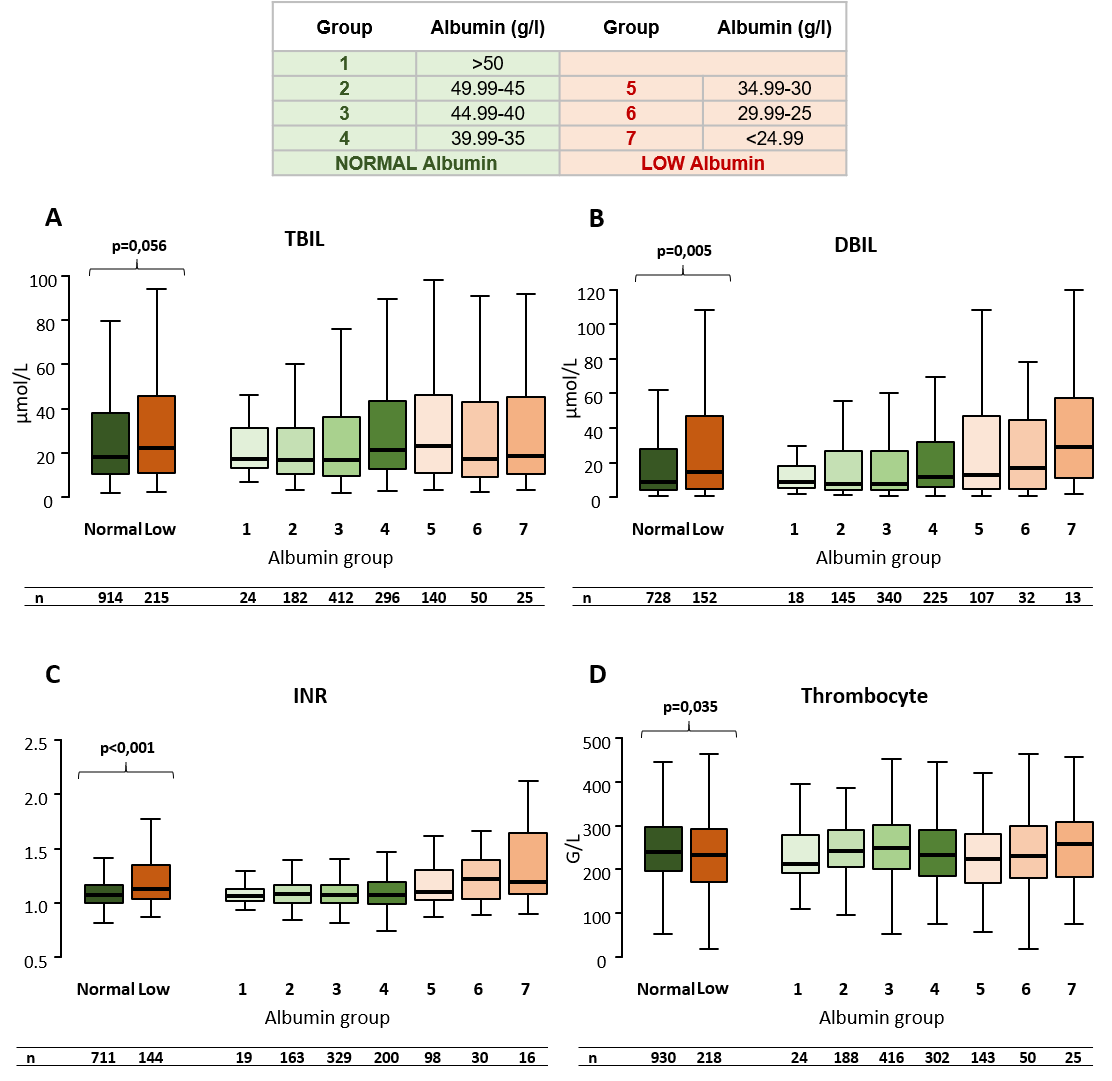


# Figure S12 – Laboratory parameters indicating liver function without outliers II.


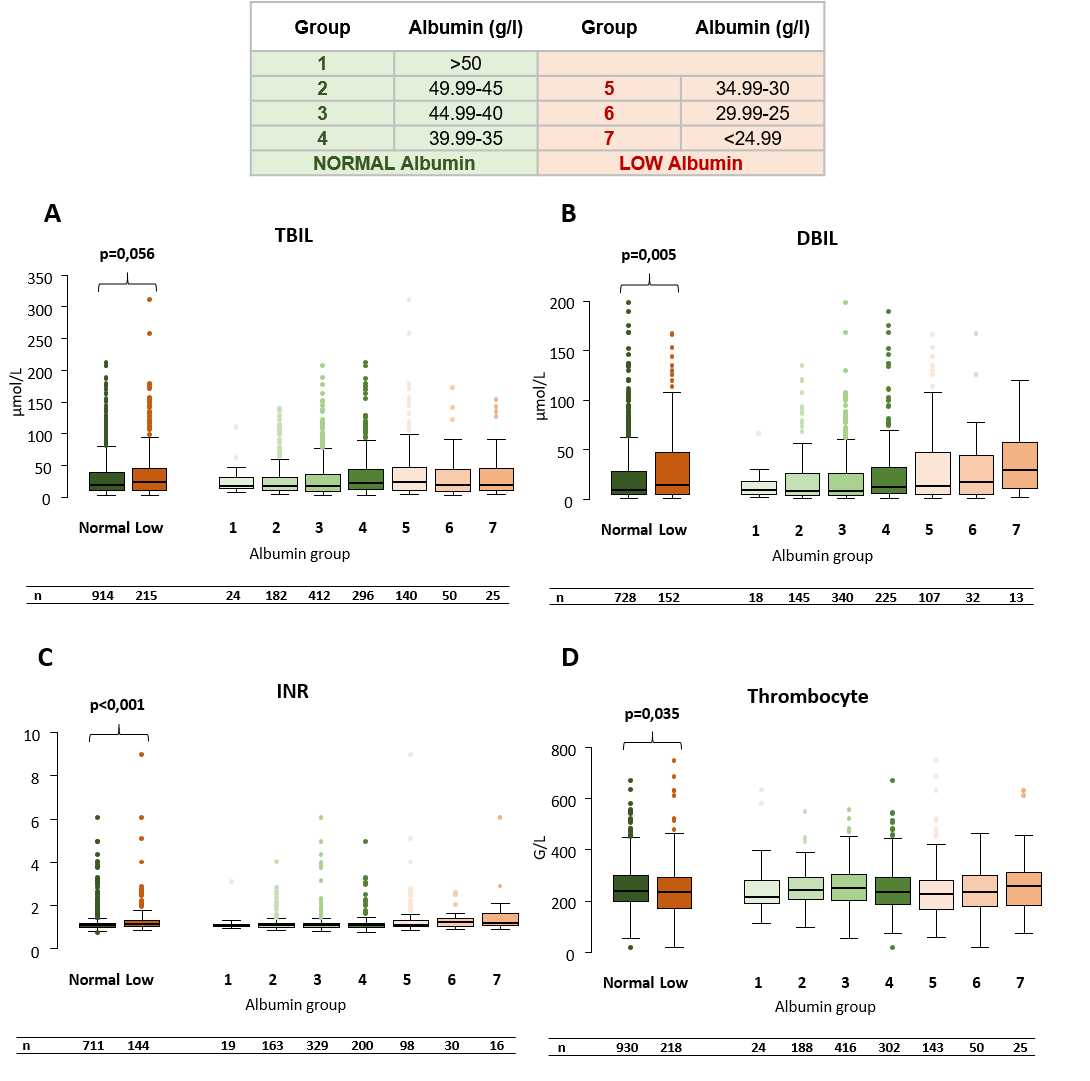


# Figure S13 – Laboratory parameters indicating liver function with outliers II.


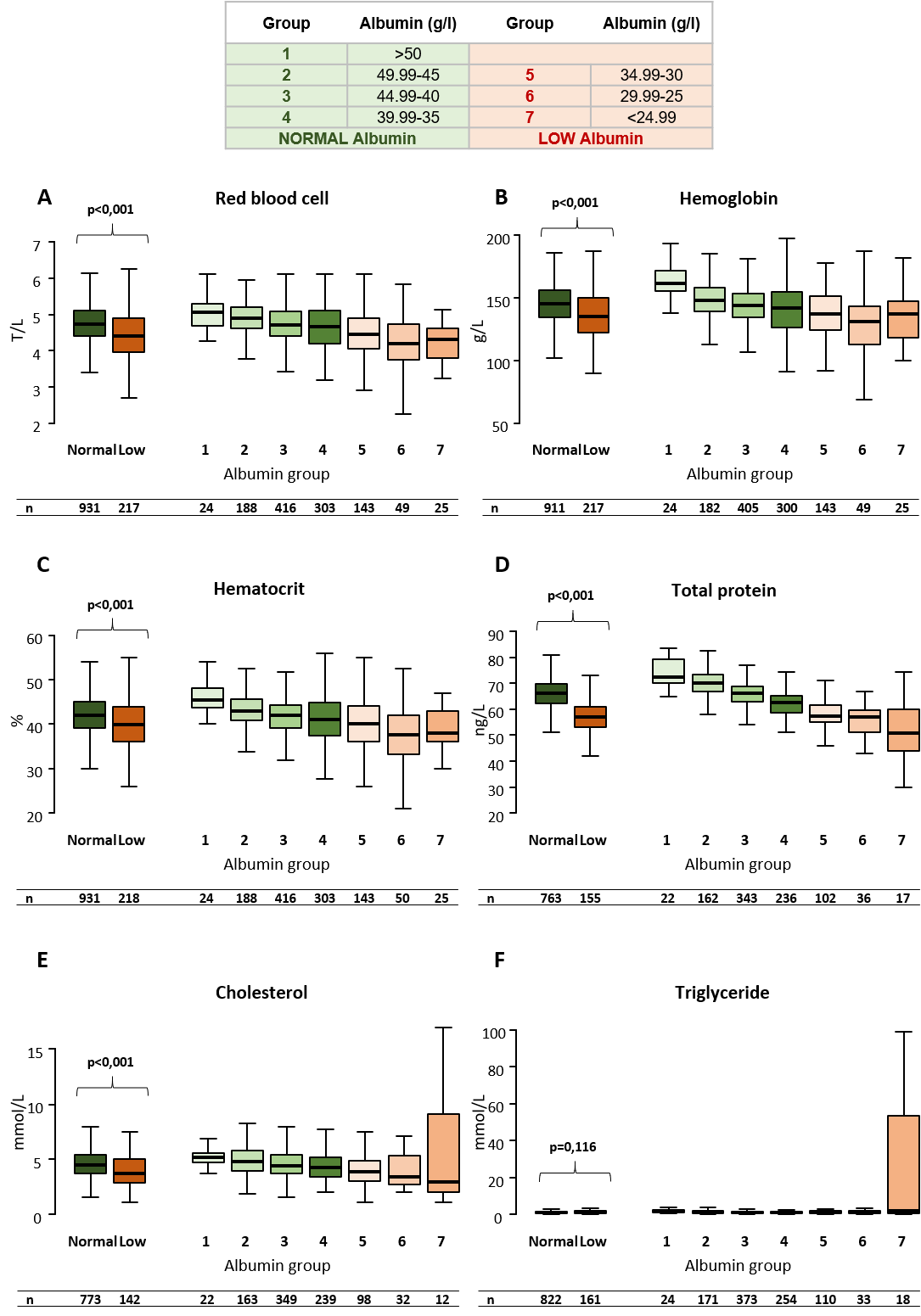


# Figure S14 – Haematological parameters and lipid levels without outliers


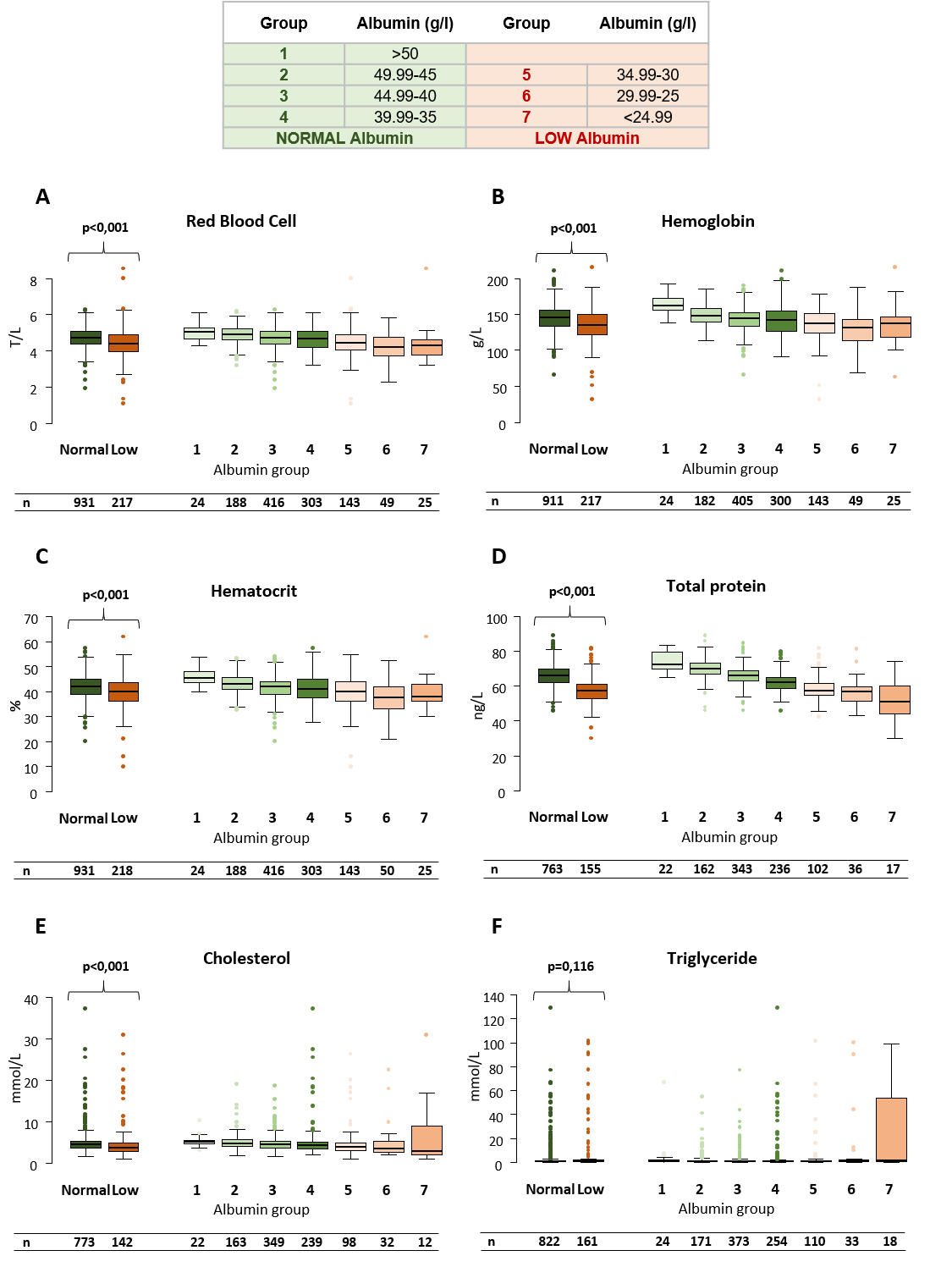


# Figure S15 – Haematological parameters and lipid levels with outliers


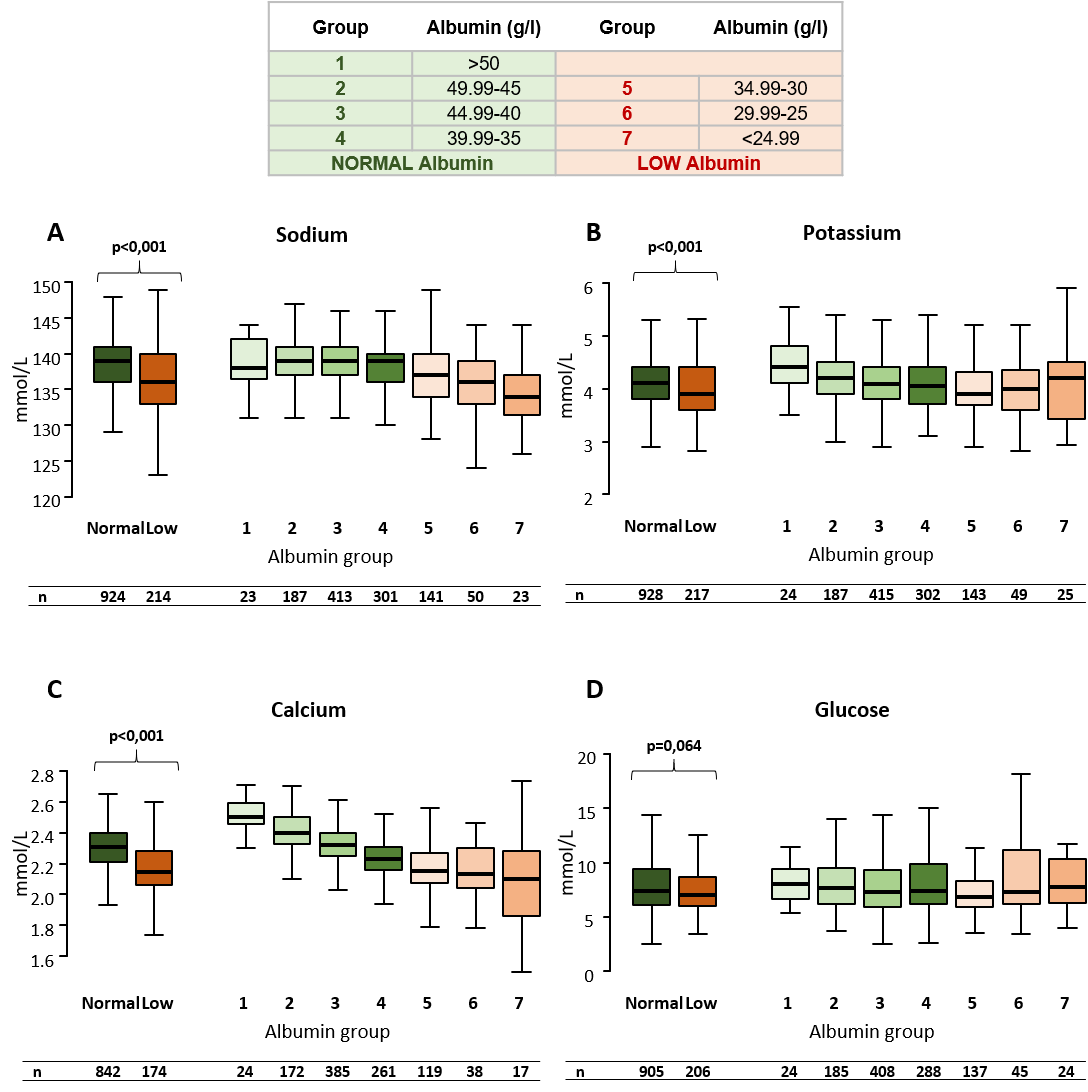


# Figure S16 – Ions and glucose levels without outliers


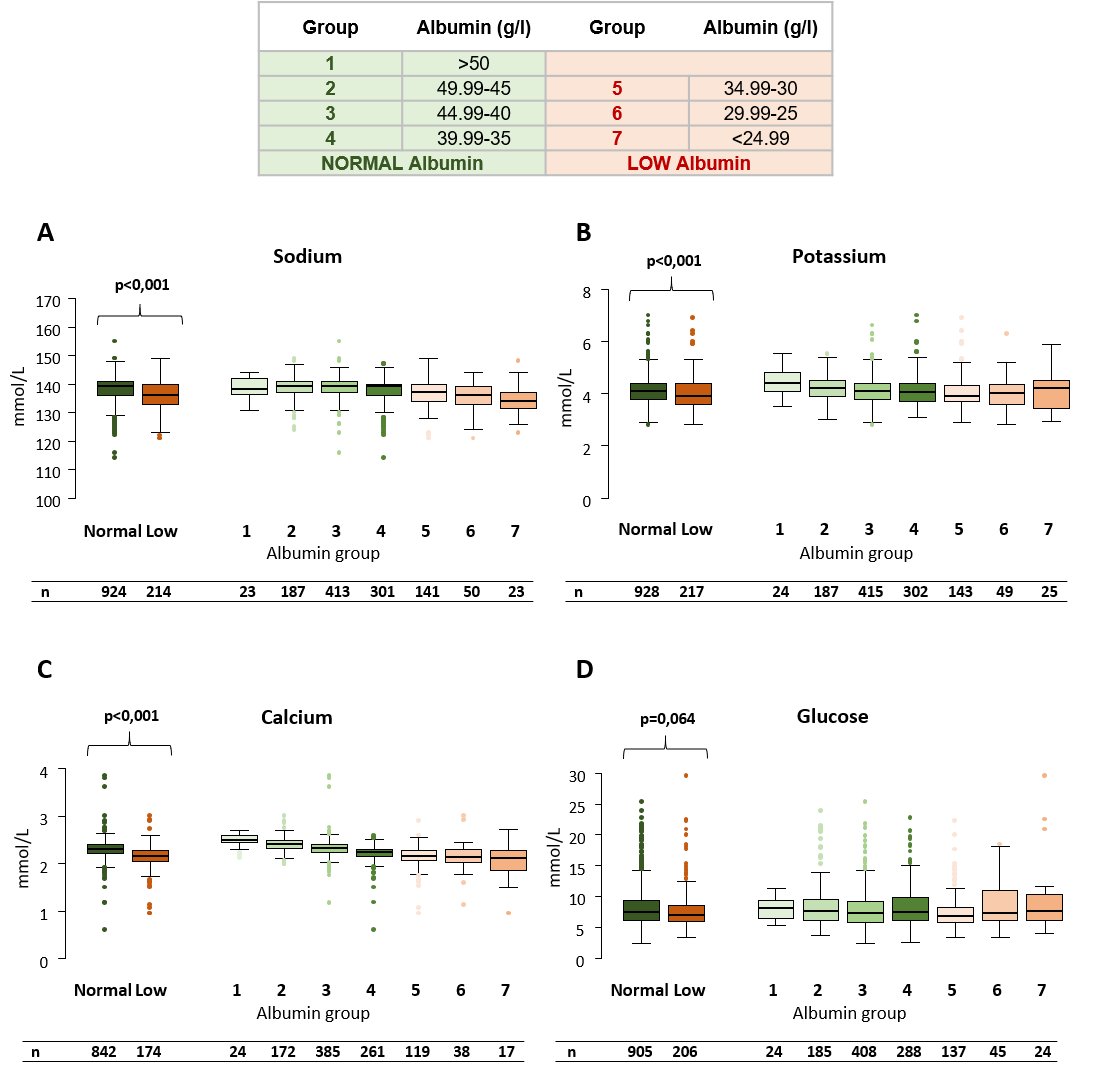


# Figure S17 – Ions and glucose levels with outliers

| On-admission albumin (n=1149) - mortality | | | | | | |
| --- | --- | --- | --- | --- | --- | --- |
| Predictor | | β | SE | OR | 95% CI | p |
| **Albumin** | <35g/L (v. ≥35 g/L) | 0.728 | 0.348 | 2.070 | 1.021-4.033 | **0.036** |
| **Age** | per years | 0.036 | 0.012 | 1.037 | 1.013-1.062 | **0.003** |
| **Gender** | female (vs. male) | -0.244 | 0.368 | 0.783 | 0.376-1.607 | 0.507 |
| **Etiology** | alcohol (vs. biliary) | 0.683 | 0.549 | 1.981 | 0.651-5.755 | 0.213 |
|  | HTG (vs. biliary) | 1.836 | 0.728 | 6.271 | 1.271-24.048 | **0.012** |
|  | biliary + alcohol (vs. biliary) | 1.029 | 1.100 | 2.797 | 0.145-16.902 | 0.350 |
|  | biliary + HTG (vs. biliary) | -13.032 | 778.318 | - | - | 0.987 |
|  | alcohol + HTG (vs. biliary) | 1.860 | 0.748 | 6.422 | 1.263-25.800 | **0.013** |
|  | idiopathic (vs. biliary) | 1.196 | 0.424 | 3.308 | 1.447-7.759 | **0.005** |
|  | other (vs. biliary) | -0.085 | 0.787 | 0.919 | 0.139-3.581 | 0.914 |
| On-admission albumin (n=1149) - severity | | | | | | |
| Predictor | | β | SE | OR | 95% CI | p |
| **Albumin** | <35g/L (v. ≥35 g/L) | 0.471 | 0.280 | 1.602 | 0.909-2.735 | 0.092 |
| **Age** | per years | 0.039 | 0.009 | 1.040 | 1.022-1.060 | **<0.001** |
| **Gender** | female (vs. male) | -0.190 | 0.280 | 0.827 | 0.475-1.429 | 0.496 |
| **Etiology** | alcohol (vs. biliary) | 0.525 | 0.401 | 1.690 | 0.760-3.670 | 0.190 |
|  | HTG (vs. biliary) | 1.818 | 0.538 | 6.160 | 2.008-17.046 | **<0.001** |
|  | biliary + alcohol (vs. biliary) | 0.953 | 0.801 | 2.594 | 0.385-10.416 | 0.234 |
|  | biliary + HTG (vs. biliary) | -13.875 | 779.136 | - | - | 0.986 |
|  | alcohol + HTG (vs. biliary) | 1.384 | 0.621 | 3.991 | 1.040-12.548 | **0.026** |
|  | idiopathic (vs. biliary) | 0.580 | 0.328 | 1.785 | 0.928-3.378 | 0.077 |
|  | other (vs. biliary) | -0.526 | 0.628 | 0.591 | 0.137-1.757 | 0.402 |

# Table S3 – Logistic regression for on-admission albumin cohort

HTG: hypertriglyceridemia; SE: standard error; OR: odds ratio; CI: confidence interval

p<0.05 was considered statistically significant (highlighted in bold)


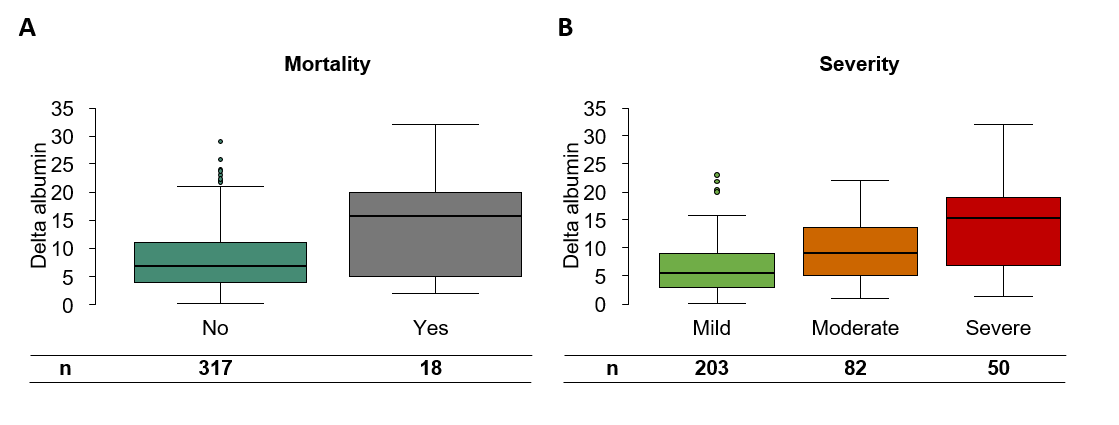


# Figure S18 – Albumin loss

| Lowest measured albumin (n=1272) – mortality | | | | | | |
| --- | --- | --- | --- | --- | --- | --- |
| Predictor | | β | SE | OR | 95% CI | p |
| **Albumin** | <35g/L (v. ≥35 g/L) | 1.431 | 0.319 | 4.185 | 2.286-8.039 | **<0.001** |
| **Age** | per years | 0.040 | 0.011 | 1.041 | 1.019-1.065 | **<0.001** |
| **Gender** | female (vs. male) | -0.420 | 0.327 | 0.657 | 0.342-1.241 | 0.200 |
| **Etiology** | alcohol (vs. biliary) | 0.972 | 0.499 | 2.642 | 0.982-7.060 | 0.052 |
|  | HTG (vs. biliary) | 1.433 | 0.723 | 4.192 | 0.855-15.870 | **0.048** |
|  | biliary + alcohol (vs. biliary) | 1.867 | 0.731 | 6.469 | 1.303-24.945 | **0.011** |
|  | biliary + HTG (vs. biliary) | -12.872 | 758.351 | - | - | 0.986 |
|  | alcohol + HTG (vs. biliary) | 1.665 | 0.741 | 5.283 | 1.049-20.864 | **0.025** |
|  | idiopathic (vs. biliary) | 1.478 | 0.383 | 4.385 | 2.104-9.572 | **<0.001** |
|  | other (vs. biliary) | -0.107 | 0.785 | 0.898 | 0.136-3.482 | 0.892 |
| Lowest measured albumin (n=1272) - severity | | | | | | |
| Predictor | | β | SE | OR | 95% CI | p |
| **Albumin** | <35g/L (v. ≥35 g/L) | 2.367 | 0.292 | 10.664 | 6.188-19.614 | **<0.001** |
| **Age** | per years | 0.029 | 0.008 | 1.029 | 1.013-1.047 | **<0.001** |
| **Gender** | female (vs. male) | -0.409 | 0.251 | 0.665 | 0.404-1.084 | 0.104 |
| **Etiology** | alcohol (vs. biliary) | 0.216 | 0.370 | 1.241 | 0.594-2.548 | 0.559 |
|  | HTG (vs. biliary) | 0.986 | 0.513 | 2.681 | 0.931-7.113 | 0.054 |
|  | biliary + alcohol (vs. biliary) | 0.549 | 0.698 | 1.732 | 0.366-6.155 | 0.431 |
|  | biliary + HTG (vs. biliary) | -13.938 | 727.870 | - | - | 0.985 |
|  | alcohol + HTG (vs. biliary) | 0.883 | 0.588 | 2.417 | 0.701-7.284 | 0.133 |
|  | idiopathic (vs. biliary) | 0.641 | 0.291 | 1.900 | 1.072-3.362 | **0.027** |
|  | other (vs. biliary) | -0.378 | 0.515 | 0.685 | 0.224-1.744 | 0.464 |

# Table S4 – Logistic regression for lowest measured albumin cohort

HTG: hypertriglyceridemia; SE: standard error; OR: odds ratio; CI: confidence interval

p<0.05 was considered statistically significant (highlighted in bold)

|  | **EPIDEMIOLOGY, ETIOLOGY** | **OVERALL** | **UPLOADED DATA** | **%** |
| --- | --- | --- | --- | --- |
| 1 | Age | 1149 | 1149 | 100% |
| 2 | Age | 1272 | 1272 | 100% |
| 3 | Gender | 1149 | 1149 | 100% |
| 4 | Gender | 1272 | 1272 | 100% |
| 5 | Etiology | 1149 | 1149 | 100% |
| 6 | Etiology | 1272 | 1272 | 100% |
|  | *Average uploaded data* | *7263* | *7263* | *100%* |
|  |  |  |  |  |
|  | **SYMPTOMS AND PHYSICAL EXAMINATION ON ADMISSION** | **OVERALL** | **UPLOADED DATA** | **%** |
| 7 | Abdominal pain | 1149 | 1148 | 100% |
| 8 | Duration of abdominal pain before admission | 1149 | 702 | 61% |
| 9 | Intensity of abdominal pain | 1149 | 1057 | 92% |
| 10 | Nausea | 1149 | 1140 | 99% |
| 11 | Vomiting | 1149 | 1141 | 99% |
| 12 | Abdominal tenderness | 1149 | 1135 | 99% |
| 13 | Abdominal guarding | 1149 | 1133 | 99% |
| 14 | Blood pressure – systolic | 1149 | 1141 | 99% |
| 15 | Blood pressure – diastolic | 1149 | 1118 | 97% |
| 16 | Heart rate | 1149 | 1130 | 98% |
|  | *Average uploaded data* | *11490* | *10845* | *94%* |
|  |  |  |  |  |
|  | **LABORATORY PARAMETERS ON ADMISSION** | **OVERALL** | **UPLOADED DATA** | **%** |
| 17 | Amylase | 1149 | 1146 | 100% |
| 18 | Lipase | 1149 | 1075 | 94% |
| 19 | Triglyceride (TG) | 1149 | 983 | 86% |
| 20 | Total cholesterol | 1149 | 915 | 80% |
| 21 | Procalcitonin | 1149 | 761 | 66% |
| 22 | C-reactive protein (CRP) | 1149 | 1138 | 99% |
| 23 | Maximum C-reactive protein (CRP) | 1172 | 1171 | 100% |
| 24 | White blood cell count (WBC) | 1149 | 1147 | 100% |
| 25 | Red blood cell count (RBC) | 1149 | 1148 | 100% |
| 26 | Hematocrit | 1149 | 1149 | 100% |
| 27 | Hemoglobin | 1149 | 1128 | 98% |
| 28 | Thrombocyte | 1149 | 1148 | 100% |
| 29 | Glucose | 1149 | 1111 | 97% |
| 30 | Aspartate transaminase (ASAT) | 1149 | 1117 | 97% |
| 31 | Alanine transaminase (ALAT) | 1149 | 1110 | 97% |
| 32 | Gamma-glutamyl transferase (γGT) | 1149 | 1104 | 96% |
| 33 | Alkaline phosphatase (ALP) | 1149 | 1125 | 98% |
| 34 | Lactate dehydrogenase (LDH) | 1149 | 1085 | 94% |
| 35 | Total bilirubin | 1149 | 1129 | 98% |
| 36 | Potassium | 1149 | 1145 | 100% |
| 37 | Sodium | 1149 | 1138 | 99% |
| 38 | Calcium | 1149 | 1016 | 88% |
| 39 | Albumin | 1149 | 1149 | 100% |
| 40 | Total protein | 1149 | 918 | 80% |
| 41 | Estimated glomerular filtration rate (eGFR) | 1149 | 1128 | 98% |
| 42 | Creatinine | 1149 | 1146 | 100% |
| 43 | Blood urea nitrogen (BUN) | 1149 | 1140 | 99% |
|  | *Average uploaded data* | *31046* | *29470* | *95%* |
|  |  |  |  |  |
|  | **OUTCOMES** | **OVERALL** | **UPLOADED DATA** | **%** |
| 44 | Local pancreatic complications | 1149 | 1135 | 99% |
| 45 | Local pancreatic complications | 1272 | 1258 | 99% |
| 46 | Peripancreatic fluid collection | 1149 | 1135 | 99% |
| 47 | Peripancreatic fluid collection | 1272 | 1258 | 99% |
| 48 | Pancreatic pseudocyst | 1149 | 1135 | 99% |
| 49 | Pancreatic necrosis | 1149 | 1134 | 99% |
| 50 | Pancreatic necrosis | 1272 | 1257 | 99% |
| 51 | Diabetes mellitus as complication | 1149 | 1149 | 100% |
| 52 | Diabetes mellitus as complication | 1272 | 1272 | 100% |
| 53 | Organ failure | 1149 | 1149 | 100% |
| 54 | Organ failure | 1272 | 1271 | 100% |
| 55 | Renal failure | 1149 | 1149 | 100% |
| 56 | Renal failure | 1272 | 1270 | 100% |
| 57 | Heart failure | 1149 | 1149 | 100% |
| 58 | Heart failure | 1272 | 1270 | 100% |
| 59 | Respiratory failure | 1149 | 1148 | 100% |
| 60 | Respiratory failure | 1272 | 1269 | 100% |
| 61 | Length of hospitalization | 1149 | 1149 | 100% |
| 62 | Length of hospitalization | 1272 | 1272 | 100% |
| 63 | Severity (mild/moderately severe/severe) | 1149 | 1149 | 100% |
| 64 | Severity (mild/moderately severe/severe) | 1272 | 1272 | 100% |
| 65 | Mortality | 1149 | 1149 | 100% |
| 66 | Mortality | 1272 | 1272 | 100% |
|  | *Average uploaded data* | *27780* | *27671* | *100%* |
|  |  |  |  |  |
|  | ***TOTAL*** | ***77579*** | ***75249*** | ***97%*** |

# Table S5 – Data quality for assessed variables

| Parameter | Normal vs Low | Normal vs Group 5 | Normal vs Group 6 | Normal vs Group 7 |
| --- | --- | --- | --- | --- |
| Figure 1 | | | | |
| Local pancreatic complications –  on-admission cohort | **0.016** | 0.553 | 0.126 | **<0.001** |
| Peripancreatic fluid collection –  on-admission cohort | **<0.001** | 0.0734 | **0.0052** | **<0.001** |
| Pancreatic necrosis –  on-admission cohort | 0.378 | 1.000 | 0.314 | 0.084 |
| Pancreatic pseudocyst –  on-admission cohort | 0.315 | 0.393 | 0.234 | 1.000 |
| Local pancreatic complications –  lowest albumin cohort | **<0.001** | **<0.001** | **<0.001** | **<0.001** |
| Peripancreatic fluid collection –  lowest albumin cohort | **<0.001** | **<0.001** | **<0.001** | **<0.001** |
| Pancreatic necrosis –  lowest albumin cohort | **<0.001** | **0.020** | **<0.001** | **<0.001** |
| Pancreatic pseudocyst –  lowest albumin cohort | **<0.001** | **0.005** | **0.001** | **<0.001** |
| Figure 2 | | | | |
| Organ failure – on-admission cohort | **<0.001** | 0.880 | **<0.001** | **<0.001** |
| Respiratory failure – on-admission cohort | 0.051 | 0.698 | **0.001** | **0.019** |
| Heart failure – on-admission cohort | 0.088 | 0.604 | 0.068 | **0.040** |
| Renal failure – on-admission cohort | **0.004** | 0.339 | **<0.001** | 0.232 |
| Organ failure – lowest albumin cohort | **<0.001** | **0.002** | **<0.001** | **<0.001** |
| Respiratory – lowest albumin cohort | **<0.001** | 0.084 | **<0.001** | **<0.001** |
| Heart failure – lowest albumin cohort | **<0.001** | 1.000 | **<0.001** | **<0.001** |
| Renal failure – lowest albumin cohort | **<0.001** | **0.045** | **<0.001** | **<0.001** |
| Figure 3 | | | | |
| Severity – on-admission cohort | **0.015** | 0.755 | 0.028 | **<0,001** |
| Mortality – on-admission cohort | **0.020** | 1.000 | **0.005** | **0.007** |
| Length of stay – on-admission cohort | **0.025** | 0.592 | 0.394 | 0.055 |
| Maximum C-reactive protein –  on-admission cohort | **<0.001** | **0.003** | **0.007** | **<0.001** |
| Severity – lowest albumin cohort | **<0.001** | **<0.001** | **<0.001** | **<0.001** |
| Mortality – lowest albumin cohort | **<0.001** | **0.784** | **0.004** | **<0.001** |
| Length of stay – lowest albumin cohort | **<0.001** | **<0.001** | **<0.001** | **<0.001** |
| Maximum C-reactive protein –  lowest albumin cohort | **<0.001** | **<0.001** | **<0.001** | **<0.001** |

| Figure S2 | | |
| --- | --- | --- |
| Representativity | Total vs on-admission | Total vs lowest measured |
| Gender | 0.964 | 0.992 |
| Severity | 0.021 | 0.005 |
| Mortality | 0.302 | 0.026 |
| Length of hospitalization | <0.001 | <0.001 |
| Age - Male | 0.563 | 0.324 |
| Age - Female | 0.853 | 0.638 |
| Age - Total | 0.591 | 0.310 |

| Parameter | Normal vs Low | Normal vs Group 5 | Normal vs Group 6 | Normal vs Group 7 |
| --- | --- | --- | --- | --- |
| Figure S3 | | | | |
| Age – on-admission cohort | 0.005 | 0.007 | 0.136 | 0.431 |
| Etiology: biliary vs non-biliary –  on-admission cohort | 0.042 | - | - | - |
| Etiology: alcohol vs non-alcohol –  on-admission cohort | 0.096 | - | - | - |
| Etiology: HTG vs non-HTG –  on-admission cohort | 0.903 | - | - | - |
| Figure S4 | | | | |
| Obesity (body mass index) | 0.012 | 0.174 | 0.025 | 0.915 |
| Diabetes mellitus – on-admission cohort |  |  |  |  |
| AP – on-admission cohort |  |  |  |  |
| RAP – on-admission cohort |  |  |  |  |
| CP – lowest albumin cohort |  |  |  |  |
| Figure 3 | | | | |
| Severity – on-admission cohort | 0.015 | 0.755 | 0.028 | <0,001 |
| Mortality – on-admission cohort | 0.020 | 1.000 | 0.005 | 0.007 |
| Length of stay – on-admission cohort | 0.025 | 0.592 | 0.394 | 0.055 |
| Maximum C-reactive protein –  on-admission cohort | <0.001 | 0.003 | 0.007 | <0.001 |
| Severity – lowest albumin cohort | <0.001 | <0.001 | <0.001 | <0.001 |
| Mortality – lowest albumin cohort | <0.001 | 0.784 | 0.004 | <0.001 |
| Length of stay – lowest albumin cohort | <0.001 | <0.001 | <0.001 | <0.001 |
| Maximum C-reactive protein –  lowest albumin cohort | <0.001 | <0.001 | <0.001 | <0.001 |

# Table S6 – Results of statistical comparisons presented on figures
